# Layered-rocksalt intergrown cathode for high-capacity zero-strain battery operation

**DOI:** 10.1038/s41467-021-22527-z

**Published:** 2021-04-20

**Authors:** Ning Li, Meiling Sun, Wang Hay Kan, Zengqing Zhuo, Sooyeon Hwang, Sara E. Renfrew, Maxim Avdeev, Ashfia Huq, Bryan D. McCloskey, Dong Su, Wanli Yang, Wei Tong

**Affiliations:** 1 Energy Storage and Distributed Resources Division, Lawrence Berkeley National Laboratory, Berkeley, CA 94720 USA; 2 Dongguan Neutron Science Center, Dongguan, Guangdong 523803 China; 3 Advanced Light Source, Lawrence Berkeley National Laboratory, Berkeley, CA 94720 USA; 4 Center for Functional Nanomaterials, Brookhaven National Laboratory, Upton, NY 11973 USA; 5 Department of Chemical and Biomolecular Engineering, University of California, Berkeley, CA 94720 USA; 6 Australian Nuclear Science and Technology Organisation (ANSTO), Lucas Heights, New South Wales 2234 Australia; 7 Neutron Scattering Science Directorate, Oak Ridge National Laboratory, Oak Ridge, Tennessee 37831 United States

**Keywords:** Batteries, Batteries

## Abstract

The dependence on lithium-ion batteries leads to a pressing demand for advanced cathode materials. We demonstrate a new concept of layered-rocksalt intergrown structure that harnesses the combined figures of merit from each phase, including high capacity of layered and rocksalt phases, good kinetics of layered oxide and structural advantage of rocksalt. Based on this concept, lithium nickel ruthenium oxide of a main layered structure (*R*$\bar{3}$*m*) with intergrown rocksalt (*Fm*$\bar{3}$*m*) is developed, which delivers a high capacity with good rate performance. The interwoven rocksalt structure successfully prevents the anisotropic structural change that is typical for layered oxide, enabling a nearly zero-strain operation upon high-capacity cycling. Furthermore, a design principle is successfully extrapolated and experimentally verified in a series of compositions. Here, we show the success of such layered-rocksalt intergrown structure exemplifies a new battery electrode design concept and opens up a vast space of compositions to develop high-performance intergrown cathode materials.

## Abstract (web summary)

The dependence on lithium-ion batteries leads to a pressing demand for advanced cathode materials. Here the authors report a new concept of layered-rocksalt intergrown structure that enables nearly zero-strain operation upon high-capacity cycling, offering tremendous opportunities to design new cathodes.

## Introduction

The increasing demand for rechargeable lithium-ion batteries of high energy and power density facilitates the continuous search for even better battery electrodes, of which cathode appears to be a key limiting factor^1,2^. Commercially viable layered oxide cathodes (e.g., LiCoO_2_ and its variants (LiNi_1-*x*-y_Mn_*x*_Co_*y*_O_2_, LiNi_1-*x*-*y*_Co_*x*_Al_*y*_O_2_, 0 < *x*, *y* < 0)) operate predominantly based on the oxidizable transition metal (TM) and extractable Li^+^ hosted in the close-packed oxygen sublattice^3–7^. These layered compounds typically exhibit high rate capability; however, they are still incapable of delivering their theoretical capacity because of the irreversible structural change at highly delithiated states^8,9^. In contrast, Li-rich metal oxides of cation-ordered (layered) and disordered rocksalt can consistently deliver a high reversible capacity of 250–300 mAh g^−1^, based on combined cationic TM and anionic oxygen redox^10–23^. However, Li-rich layered oxide cathode suffers from an irreversible layered-to-spinel/rocksalt phase transformation, accompanied by lattice oxygen loss, leading to severe capacity and voltage decay upon electrochemical cycling^24–28^. Mitigating these effects for practical application remains a formidable challenge^29–35^. Li-excess disordered rocksalt, although with a minimal isotropic structural change upon (de)lithiation, needs to be pulverized to nanoscale and cycled at low currents^23,36,37^.

Layered and rocksalt structure share a similar close-packed oxygen framework, but with different arrangements in Li and TM: layered structure exhibits cation ordering between alternating Li and TM slabs, whereas Li and TM are mostly randomly distributed in the disordered rocksalt. Therefore, it is principally possible to develop a material that integrates the favored structural and electrochemical attributes of both layered and rocksalt structures. So far, very limited success has been achieved to utilize the structural compatibility of layered and rocksalt phases for the development of high-performance Li-ion cathodes^38–40^. Herein, we propose and demonstrate a concept of layered-rocksalt intergrown structure for the development of advanced Li-ion cathode, which is intrinsically different from the well-known Li/TM intermixing or the formation of densified surface phase in layered cathode during synthesis or upon electrochemical cycling (Supplementary Fig. 1). Such a layered-rocksalt intergrown structure harnesses the favored figures of merit from each individual component as follows: (1) the inherently high capacity of layered and rocksalt phases; (2) good kinetics (rate capability) from the facile Li^+^ diffusion in the layered oxide; and (3) isotropic structural change with largely reduced mechanical stress benefiting from the interwoven rocksalt phase.

In this work, we have successfully designed and synthesized lithium nickel ruthenium oxides based on Ni^2+^/Ru^5+^ combination, Li_1.2_Ni_0.4_Ru_0.4_O_2_, which exhibits a main layered structure (*R*$\bar{3}$*m*) with well-grown rocksalt (*Fm*$\bar{3}$*m*) nanodomains. Li_1.2_Ni_0.4_Ru_0.4_O_2_ delivers a high reversible capacity of 240–330 mAh g^−1^ with good rate capability. We unravel an intriguing isotropic structural evolution with a negligible change in crystal lattice upon Li^+^ (de)insertion, resembling that of the disordered rocksalt. We also verify that the design of such intergrown structure requires TM with appropriately selected ionic radius and/or valence state, as well as electronic configuration. As both phases accommodate a vast composition space, given the excellent tolerance for stoichiometry and TM combination in layered and disordered rocksalts^41^, our demonstration opens up immense opportunities in developing high-performance intergrown electrode materials.

## Results

### Layered-rocksalt intergrown structure

**Fig. 1:**
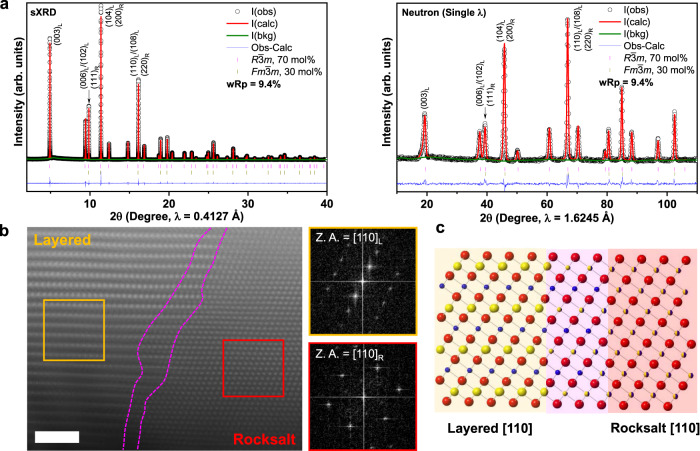
Li_1.2_Ni_0.4_Ru_0.4_O_2_ of layered-rocksalt intergrown structure. **a** sXRD and ND patterns with Rietveld fits for Li_1.2_Ni_0.4_Ru_0.4_O_2_; refinement is performed based on *R*$\bar{3}$*m* and *Fm*$\bar{3}$*m* biphasic model, indicating 70 mol% *R*$\bar{3}$*m* (*a* = 2.94194(7) Å, *c* = 14.40729(2) Å, and *V* = 107.990(1) Å^3^) and 30 mol% *Fm*$\bar{3}$*m* (*a* = 4.15874(6) Å and *V* = 71.926(1) Å^3^); critical reflections are indexed as (hkl)_L_ and (hkl)_R_ for *R*$\bar{3}$*m* and *Fm*$\bar{3}$*m*, respectively. **b** Representative HAADF-STEM image and FFT of the selected areas highlighted by orange and red box, respectively, showing the layered-rocksalt intergrown structure along the [110] zone axis. Scale bar is 2 nm. **c** Schematic of layered-rocksalt intergrown structure along the [110] zone axis, pink highlight showing the structurally compatible region with different TM arrangements in the Li and TM slabs.

Li_1.2_Ni_0.4_Ru_0.4_O_2_ was prepared by a solid-state reaction and the crystal structure at pristine state is carefully examined by a joint synchrotron X-ray diffraction (sXRD) and neutron diffraction (ND). All the reflections in Fig. 1a can be well indexed, based on layered *R*$\bar{3}$*m* structure. In particular, no super-lattice peaks in the 2*θ* region of 5°–9° (*λ* = 0.4127 Å), originating from the Li/TM ordering in the TM slabs, are noticed in sXRD of Li_1.2_Ni_0.4_Ru_0.4_O_2_. Rietveld refinement of sXRD for pristine Li_1.2_Ni_0.4_Ru_0.4_O_2_ based on *R*$\bar{3}$*m* space group leads to a good fit in the peak position. However, a discrepancy in the peak intensity is revealed, especially for the intensity ratio of reflection (003)/(104), which is an important indicator of the degree of cation ordering in layered *R*$\bar{3}$*m* phase (Supplementary Fig. 2). Low intensity ratio of reflection (003)/(104) in layered *R*$\bar{3}$*m* could be due to Li/Ni intermixing because of the similar ionic radius of Li^+^ (0.76 Å) and Ni^2+^ (0.69 Å)^42–45^. Simulation of XRD patterns based on *R*$\bar{3}$*m* clearly shows the decreased intensity of (003) reflection with respect to (104) reflection when the level of Li/Ni mixing increases (Supplementary Fig. 3). Additional simulated XRD and ND patterns for *R*$\bar{3}$*m* and *Fm*$\bar{3}$*m* are presented in Supplementary Fig. S4. Therefore, further joint refinement of sXRD and ND is performed based on single *R*$\bar{3}$*m* phase model with Li/Ni intermixing (Supplementary Fig. 5). Single *R*$\bar{3}$*m* phase model that allows Li/Ni intermixing results in a better fit in peak intensity with a final *R*-factor of 13.8%, revealing ~8.0% Li/Ni intermixing. Meanwhile, close comparison of the calculated and observed XRD patterns reveals the deviation of the reflections around 9.9°, 11.4°, and 16.1° (*λ* = 0.4127 Å), which are in accordance with the characteristic reflections of the rocksalt phase. Furthermore, a joint refinement based on layered-rocksalt biphasic model leads to an even lower *R*-factor of 9.4% and the optimal refinement indicates the final product is composed of 70 mol% layered and 30 mol% rocksalt phase (Fig. 1a, Supplementary Fig. 6, and Supplementary Table 1).

To further verify the structure of Li_1.2_Ni_0.4_Ru_0.4_O_2_, high-angle annular dark field-scanning transmission electron microscopy (HAADF-STEM) was employed to directly visualize the atomic distribution of TMs. A number of particles are examined and a representative HAADF-STEM image is shown in Fig. 1b, with additional images from different particles presented in Supplementary Fig. 7 supporting this global feature. HAADF-STEM clearly reveals the typical layered arrangement of TMs in one domain (left) and rocksalt pattern in the other domain (right), also confirmed by fast Fourier transformation (Fig. 1b). More importantly, the layered-rocksalt components are not randomly separated in crystal grains; instead, they exhibit an intergrown structure that firmly anchors the rocksalt domain in the main layered phase. Careful examination of the boundary between layered and rocksalt domains reveals a structurally compatible region, where a gradual transition is clearly distinguished by different TM distribution in the Li and TM slabs, as opposed to a grain boundary. Electron energy loss spectroscopy (EELS) mapping of the pristine Li_1.2_Ni_0.4_Ru_0.4_O_2_ (Supplementary Fig. 8) reveals uniform elemental distribution in both rocksalt and layered regions. Therefore, our combined sXRD, ND, and STEM analysis consistently and unambiguously reveals the new layered-rocksalt intergrown structure for pristine Li_1.2_Ni_0.4_Ru_0.4_O_2_ (Fig. 1c).

### Electrochemical characterization

**Fig. 2:**
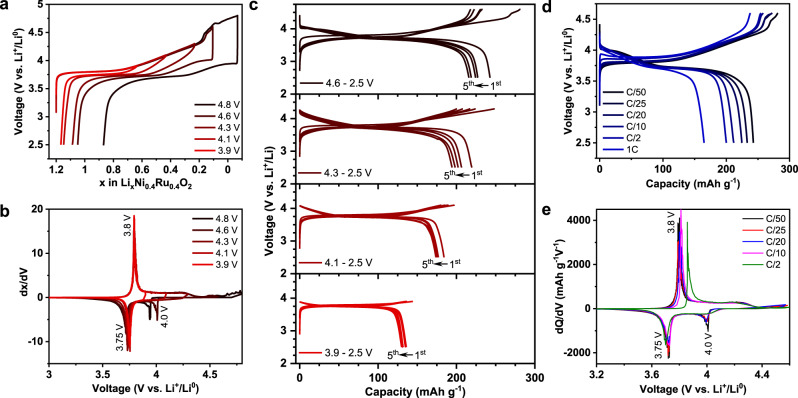
Electrochemical characterization of Li_1.2_Ni_0.4_Ru_0.4_O_2_. **a** The first cycle voltage profiles, **b** dx/dV plots, and **c** voltage profiles during the first five cycles at different charge cutoff voltages. **d** Voltage profiles and **e** dQ/dV plots at different rates. Cells are cycled at 5 mA g^−1^ in **a**–**c** and between 4.6 and 2.5 V in **d**, **e**.

Electrochemical activity of Li_1.2_Ni_0.4_Ru_0.4_O_2_ with a layered-rocksalt intergrown structure (Fig. 2) is investigated directly on the as-produced material with a particle size of ~500 nm (Supplementary Fig. 9) without further modification. It is initially subjected to galvanostatic charge and discharge testing at various charge cutoff voltages, ranging from 3.9 to 4.8 V. We reveal continuous Li^+^ extraction and increased Li^+^ uptake upon increasing charge cutoff voltage (Fig. 2a). Li_1.2_Ni_0.4_Ru_0.4_O_2_ displays the best electrochemical reversibility between 4.6 and 2.5 V, featured by ~1.1 Li^+^ extraction and 0.95 Li^+^ re-insertion (244 mAh g^−1^ and 904 Wh kg^−1^) during charge and discharge, respectively. Given the total of 1.2 Li^+^ inventory in the material, such layered-rocksalt intergrown oxide enables high % of Li^+^ extraction/insertion, which is comparable to that in Li-rich layered oxide and disordered rocksalt. No additional reversible capacity is gained beyond 4.6 V charge cutoff. Further expanding the voltage window to 4.8–1.5 V leads to a discharge capacity of 333 mAh g^−1^ (Supplementary Fig. 10). The differential capacity curves (Fig. 2b) are characterized by a sharp anodic peak around 3.8 V upon charge with a common cathodic peak around 3.75 V upon discharge, perhaps relating to Ni redox. Ni^2+^/Ru^5+^ or Ni^3+^/Ru^4+^ combination is possible in Li_1.2_Ni_0.4_Ru_0.4_O_2_. Nickel can be electrochemically active through Ni^2+^/Ni^4+^ (2*e*^−^) or Ni^3+^/Ni^4+^ (1*e*^−^), but only Ru^4+^/Ru^5+^ redox is possible for Ruthenium. In either case, TM redox can only account for 0.8 Li^+^ (206 mAh g^−1^). Interestingly, an additional cathodic peak around 4 V starts to evolve when the charge cutoff voltage reaches to 4.6 V, indicating the possible contribution of oxygen redox in the high-voltage region. Meanwhile, Li_1.2_Ni_0.4_Ru_0.4_O_2_ demonstrates better capacity retention at cutoff voltages < 4.3 V (Fig. 2c). The rate capability is also evaluated directly on Li_1.2_Ni_0.4_Ru_0.4_O_2_ at the rates ranging from C/50 to 1C between 4.6 and 2.5 V. The material delivers a discharge capacity of ~200 and 165 mAh g^−1^ at C/2 and 1C, respectively (Fig. 2d). With increasing current density, the charge and discharge profiles mostly retain, characterized by a pair of anodic/cathodic peaks around 3.75 V (Fig. 2e), whereas the cathodic peak around 4 V remains at low rates and becomes less pronounced at ≥C/10, in accordance with slightly high polarization at >4 V discharge observed by Galvanostatic intermittent titration technique (Supplementary Fig. 10). In general, the layered-rocksalt intergrown Li_1.2_Ni_0.4_Ru_0.4_O_2_ displays a high capacity and good rate capability; more importantly, it largely mitigates the notorious hysteresis of typical Li-rich layered oxides during the initial cycles.

### Isotropic and nearly zero-strain structural evolution

**Fig. 3:**
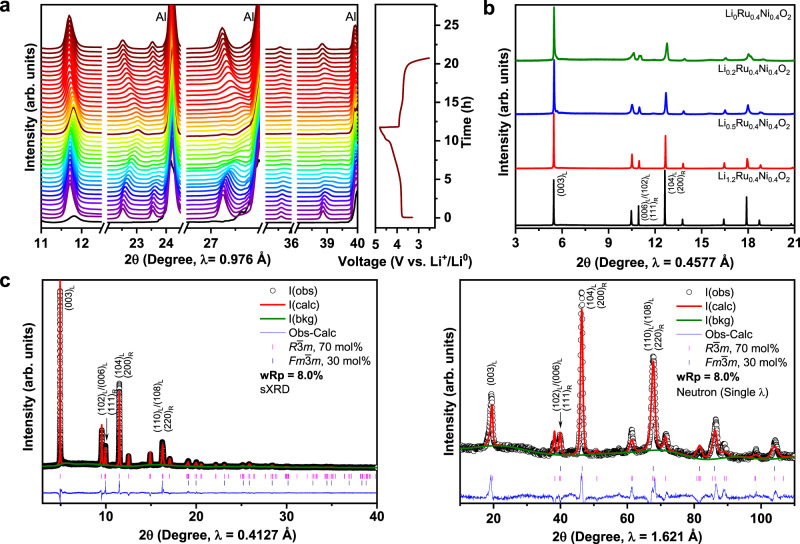
Nearly zero-strain isotropic structural evolution of Li_1.2_Ni_0.4_Ru_0.4_O_2_ upon delithiation/lithiation. **a** In situ sXRD of Li_1.2_Ni_0.4_Ru_0.4_O_2_, the black pattern at the bottom is the background from the in situ cell; cell was cycled between 4.8 and 2.5 V at C/10. **b** sXRD of Li_*x*_Ni_0.4_Ru_0.4_O_2_ (*x* = 1.2, 0.5, 0.2, 0) prepared by chemical delithiation method. **c** Joint refinement of sXRD and ND patterns of Li_0.2_Ni_0.4_Ru_0.4_O_2_. Critical reflections are indexed as (hkl)_L_ and (hkl)_R_ for *R*$\bar{3}$*m* and *Fm*$\bar{3}$*m*, respectively.

To investigate the evolution of layered-rocksalt intergrown structure upon electrochemical cycling, in situ sXRD patterns are collected on a pouch cell composed of Li_1.2_Ni_0.4_Ru_0.4_O_2_//Li between 4.8 and 2.5 V at C/10 (Fig. 3a). Here, in situ sXRD analysis mainly focuses on the general structural change upon delithiation/lithiation, because the reflections of layered *R*$\bar{3}$*m* and *Fm*$\bar{3}$*m* rocksalt partially overlap with those of in situ pouch cell. Clearly, there is no new phase formation upon electrochemical cycling. Strikingly, the lattice parameters *a* and *c* of layered *R*$\bar{3}$*m* component exhibit an isotropic change (Supplementary Fig. 11), as evidenced by all reflections shifting to a slightly higher diffraction angle upon charging and shifting back upon discharging. Such isotropic change in crystal lattice upon electrochemical cycling is further verified by ex situ sXRD collected on the cycled electrodes at different states of charge (Supplementary Fig. 12). More importantly, the layered-rocksalt intergrown structure still remains even after 100 cycles (Supplementary Fig. 13). Indeed, conventional layered oxides of *R*$\bar{3}$*m* structure experiences an anisotropic change, which is characterized by a gradual increase in *c* lattice parameter accompanied by a slight decrease in *a* lattice upon delithiation, due to the change in ionic radius of TM and repulsion between the TM slabs at different states of charge^46,47^. In sharp contrast, although with 70 mol% layered *R*$\bar{3}$*m* phase, Li_1.2_Ni_0.4_Ru_0.4_O_2_ displays an isotropic structural change, resembling that of Li-excess disordered rocksalt. Therefore, 30 mol% intergrown rocksalt can effectively manipulate the slabs of the layered matrix so that an isotropic lattice change becomes dominant upon delithiation/lithiation.

Furthermore, a series of Li_*x*_Ni_0.4_Ru_0.4_O_2_ samples at varied states of delithiation have been prepared via a chemical delithiation method for the detailed analysis of the structural evolution of each component. sXRD patterns of chemically delithiated Li_*x*_Ni_0.4_Ru_0.4_O_2_ (Fig. 3b) are consistent with those obtained from the electrochemical cells. All the characteristic reflections retain upon delithiation. Close comparison reveals a very small shift towards the higher 2*θ* angle. For Li_0.5_Ni_0.4_Ru_0.4_O_2_, the most pronounced change is the decrease in the intensity of the reflections at 10.9, 12.7, and 17.9° (*λ* = 0.4577 Å), which are characteristic of rocksalt component, suggesting the delithiation perhaps starts from *Fm*$\bar{3}$*m* rocksalt. The refinement of the ND reflection of chemically delithiated Li_0.5_Ni_0.4_Ru_0.4_O_2_ sample also indicates the preference of Li^+^ extraction from rocksalt rather than layered phase during initial Li^+^ extraction, as evidenced from the slightly higher lithium content in the *R*$\bar{3}$*m* phase than in the *Fm*$\bar{3}$*m* phase (Supplementary Fig. 14). A specific constrain is created in the refinement process (Supplementary Note 1). These results imply the two-dimensional Li^+^ diffusion channels of layered *R*$\bar{3}$*m* can help facilitate the extraction of Li^+^ from the intergrown *Fm*$\bar{3}$*m* rocksalt.

Chemically delithiated Li_0.2_Ni_0.4_Ru_0.4_O_2_ sample is further investigated, as it is close in composition to the electrode electrochemically charged to 4.6 V, with almost 1 Li^+^ extracted from Li_1.2_Ni_0.4_Ru_0.4_O_2_. Joint sXRD and ND refinements based on biphasic model generate a final *R*-factor of 8.0% (Fig. 3c and Supplementary Table 2). It is worth noting that both lattice parameter *a* and *c* of the layered *R*$\bar{3}$*m* component show an exceptionally low change of ~1%, which can be referred to as “nearly zero-strain” electrode. Moreover, the fraction of layered *R*$\bar{3}$*m* and *Fm*$\bar{3}$*m* rocksalt is consistent with that of the pristine state, suggesting the delithiation process does not alter the overall phase composition. Therefore, the layered-rocksalt intergrown phase displays an excellent structural robustness with the minimal change in lattice parameters upon delithiation/lithiation.

### Cationic TM redox mechanism

**Fig. 4:**
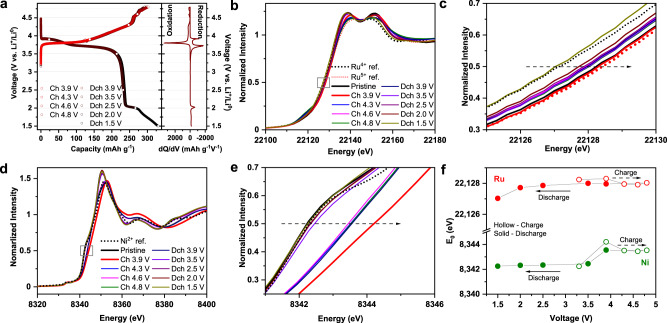
Cationic redox mechanism of Li_1.2_Ni_0.4_Ru_0.4_O_2_. **a** Voltage profiles and dQ/dV plot of Li_1.2_Ni_0.4_Ru_0.4_O_2_, showing samples (open circles) for ex situ XAS analysis. **b**, **c** XANES of Ru *K*-edge. **d**, **e** XANES of Ni *K*-edge. **f** Ni and Ru K-edge energy measured at half maxima at different states of charge.

In parallel with the structural evolution study, the oxidation states of Ni and Ru at pristine and different states of charge are probed using hard X-ray absorption spectroscopy (XAS), to determine the charge compensation mechanism of TMs. Samples that are of interest are selected for a detailed characterization based on the dQ/dV plot (Fig. 4a). From hard XAS (Fig. 4b–e), the energy of Ru and Ni at half maxima is consistent with charged Li_2_RuO_3_ (red dot line in Fig. 4b, c) and LiNi_1/3_Mn_1/3_Co_1/3_O_2_ (black dot line in Fig. 4d, e), implying Ru^5+^/Ni^2+^ combination in pristine Li_1.2_Ni_0.4_Ru_0.4_O_2_. When the electrode is charged to 3.9 V, Ru *K*-edge energy remains consistent with Ru^5+^ reference, because Ru^5+^ cannot be further oxidized, whereas the Ni *K*-edge shifts from Ni^2+^ reference to a higher energy, a clear indication of Ni oxidation. Interestingly, both Ru and Ni *K*-edge show an abnormal shift to a lower energy upon further charging to 4.3 V and beyond, up to 4.8 V, indicating the “reduction” of Ru and Ni upon charging in the high-voltage region. Ni reduction at a highly charged state is further verified by a two-dimensional transmission X-ray microscopy (TXM) measurement (Supplementary Fig. 15). These results further confirm that other oxidation reaction beyond TM such as O accounts for the Li^+^ extraction in the high-voltage region. Upon discharge, *K*-edge energy remains same until 2.0 V for Ru and 3.9 V for Ni, implying no cationic TM redox by 3.9 V. Therefore, cathodic peak at 4.0 V (Fig. 2b, e) can be unambiguously attributed to anionic O reduction. Further discharging to 1.5 V leads to Ru reduction to 4+, because Ru *K*-edge energy at 1.5 V matches that of pristine Li_2_RuO_3_ (black dot line in Fig. 4b, c). After 3.9 V discharge, Ni *K*-edge shows a significant shift to a lower energy, close to pristine 2+, which is fully recovered at 2 V, and shows no further change upon discharging to 1.5 V; therefore, Ni redox largely accounts for the anodic/cathodic peaks around 3.75 V (Fig. 2b, e). Such a trend in TM oxidation state change upon charging/discharging can be easily visualized in Fig. 4f, also confirmed by extended X-ray absorption fine structure (Supplementary Fig. 16). Overall, Ni and Ru are present as 2+ and 5+ at pristine state; Ni redox mainly accounts for cationic TM redox, whereas Ru remains inactive. We also infer that O participates in the electrochemistry in the high-voltage region, accounting for the second redox around 4 V, which will be further discussed below.

### Anionic oxygen-redox mechanism

**Fig. 5:**
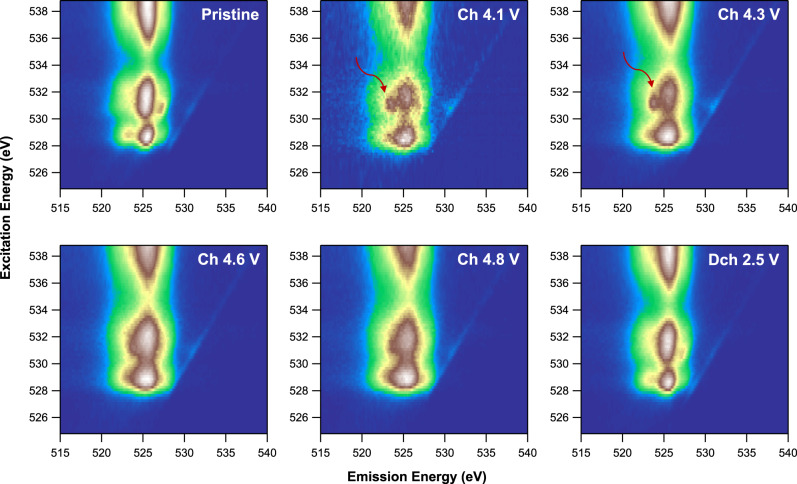
Anionic redox mechanism of Li_1.2_Ni_0.4_Ru_0.4_O_2_. O *K*-edge mRIXS results at different electrochemical states. Red arrows indicate the fingerprinting feature of oxidized oxygen at the excitation and emission energy of 531 and 523.7 eV, respectively. The feature emerges at 4.1 V during charge and disappears in the following discharge, clearly revealing a reversible lattice oxygen-redox reaction.

Both electrochemistry (Fig. 2) and TM XAS (Fig. 4) indicate that anionic oxygen participates in the electrochemistry of Li_1.2_Ni_0.4_Ru_0.4_O_2_ in the high-voltage region. We therefore perform high-efficiency mapping of resonant inelastic X-ray scattering (mRIXS) at O *K*-edge, which has been established as a reliable probe of lattice oxygen redox^48,49^. In general, the mRIXS images (Fig. 5) are dominated by three broad features around 525 eV emission energy (horizontal axis), which are typical O^2^^−^ features for oxides with excitation energies (vertical axis) of 528–533 eV and above 535 eV, corresponding to the TM-*d* and -*s*/*p* states hybridized to O*-2p* states, respectively^50^. It is noteworthy that the excitation energy here is the same as that in typical O*-K* soft XAS spectra, but mRIXS is capable of differentiating the intrinsic oxidized oxygen signals from the dominating TM characters along the new dimension of emission energy, revealing a fingerprinting feature of lattice oxygen-redox state at 523.7 eV emission energy (red arrows in Fig. 5)^48,49^. This particular mRIXS feature corresponds to the electron excitation into unoccupied O*-2p* states, thus fingerprinting the lattice oxidized oxygen, because O^2−^ has no unoccupied *2p* states^50^. This oxidized oxygen feature emerges when the Li_1.2_Ni_0.4_Ru_0.4_O_2_ electrode is charged to 4.1 V. Given about 0.8 Li^+^ is extracted from Li_1.2_Ni_0.4_Ru_0.4_O_2_ at 4.1 V charge, oxygen oxidation takes place with almost full oxidation of Ni^2+^ to Ni^4+^, consistent with our TM XAS results (Fig. 4). The intensity of the lattice O redox feature increases upon further charging, while the hybridization features along 525 eV emission energy also get enhanced due to the increasing covalency of the overall system upon charging. At 4.6 and 4.8 V charged states, the two groups of growing features get overlapped, but the oxidized oxygen feature remains clear via a direct comparison of the individual RIXS spectra cut from the mRIXS image along the 531 eV excitation energy (Supplementary Fig. 17). In addition, such oxidized lattice oxygen feature completely disappears at 2.5 V discharged state, indicating a reversible oxygen-redox reaction.

It is noteworthy that typical oxygen-redox-active Li-rich compounds always display a finite amount of broadening of the mRIXS features after discharge compared to pristine state, because of their severe structural changes during the initial cycle^17,50^. In contrast, Li_1.2_Ni_0.4_Ru_0.4_O_2_ electrode at the discharged state here recovers completely to its pristine state. Again, this is highly consistent with the robustness of such layered-rocksalt intergrown structure upon cycling. The reversible lattice oxygen redox during the charge and discharge of Li_1.2_Ni_0.4_Ru_0.4_O_2_ is further supported by the gas evolution measured by operando differential electrochemical mass spectrometry (DEMS) (Supplementary Fig. 18), showing minimal oxygen and CO_2_ gas release during the first cycle. A burst of CO_2_ evolution at 4.3 V charge mostly originates from the carbonate residual from the synthesis^51^. Therefore, we clearly revealed that the lattice oxygen redox is mostly reversible in Li_1.2_Ni_0.4_Ru_0.4_O_2_ with negligible irreversible O loss, which is of critical importance not only for practical utilization of combined cationic and anionic redox reactions but also for fundamental understanding to differentiate these two oxygen activities, i.e., lattice oxygen redox and oxygen loss^28^.

## Discussion

Materials exhibiting robust structure upon the high-capacity cycling are tremendously important for high-performance batteries. With Li_1.2_Ni_0.4_Ru_0.4_O_2_, we demonstrate a high capacity through combined TM/O redox and nearly zero-strain isotropic structural changes are enabled in layered-rocksalt intergrown structure. Based on the successful demonstration of Li_1.2_Ni_0.4_Ru_0.4_O_2_, we further explore the formation of such intergrown structure on other compositions, aiming to extrapolate the universal material design principle. We take our initial consideration based on the ionic radius of the TM. In general, ordered layered oxides are favored when the radius size of the TM cation is largely differed from that of Li^+^. Cation mixing between Li and TM tends to occur for the TM ions with a radius size similar to Li^+^ (0.76 Å), such as Ni^2+^ (0.69 Å), Mn^2+^ (0.67 Å), and Mn^3+^ (0.65 Å)^52^. In addition, the electronic structure of the TM cation also plays a critical role in the formation of ordered layered and disordered rocksalt structure^36,53,54^. The more electrons on *d* shell, the more difficult to distort electronic structure and accommodate the strain associated with rocksalt phase formation. Therefore, *d*^0^ TM with fully distortable electronic structure prefers rocksalt formation, whereas layered phase formation is more feasible for *d*^6^ TM with fixed electronic structure. For example, the early TMs with *d*^0^ orbital (e.g., Ti^4+^, Nb^5+^, and Mo^6+^), the electronic configuration of which promotes the formation of the disordered rocksalt. Therefore, the general principle to design such intergrown structure is to choose the TM cation with comparable radius size to Li^+^, combined with the TM featuring less distortable electronic configuration, to form the disordered rocksalt and ordered layered structure, respectively.

**Fig. 6:**
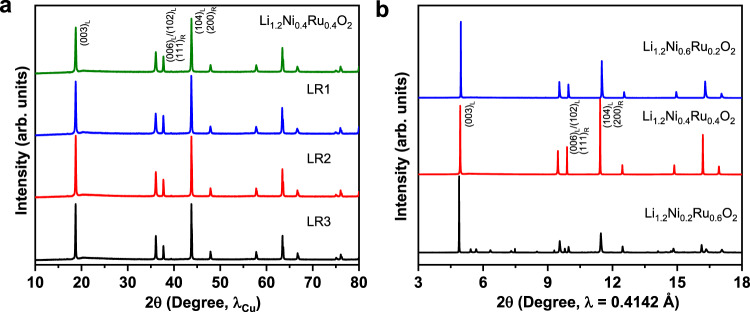
New Li-rich metal oxides of different Ni/Ru combination. **a** XRD patterns based on Ni^2+^/Ru^5+^ combination, showing layered-rocksalt intergrown structure. The designed layered-rocksalt samples Li_7/6_Ni_4/9_Ru_7/18_O_2_, Li_5/4_Ni_1/3_Ru_5/12_O_2_, and Li_4/3_Ni_2/9_Ru_4/9_O_2_ are labeled as LR1, LR2, and LR3, respectively. **b** XRD patterns based on varied oxidation states of Ni/Ru, where Ni^2+^/Ru^4+^ in Li_1.2_Ni_0.2_Ru_0.6_O_2_ and Ni^3+^/Ru^5+^ in Li_1.2_Ni_0.6_Ru_0.2_O_2_ lead to layered structure vs. layered-rocksalt intergrown structure for Ni^2+^/Ru^5+^ in Li_1.2_Ni_0.4_Ru_0.4_O_2_. Critical reflections are indexed as (hkl)_L_ and (hkl)_R_ for *R*$\bar{3}$*m* and *Fm*$\bar{3}$*m*, respectively.

In this case, the combination of Ni^2+^ (0.69 Å) and Ru^5+^ (0.57 Å, 4*d*^3^) leads to the formation of layered-rocksalt intergrown structure. Indeed, this combination of Ni^2+^ and Ru^5+^ enables the intergrown structure in a quite reasonable composition range (Fig. 6a and Supplementary Fig. 19). Rietveld refinement analysis (Supplementary Fig. 20 and Supplementary Tables 3–6) shows the molar ratio of rocksalt phase gradually increases from 20.7% to 32.6% with increasing Ni content or Ni/Ru ratio (from 0.5 to 1.14), indicating the effect of the composition on the phase ratio in the intergrown structure. The valence state of Ni and Ru in the designed samples is confirmed to be 2+ and 5+, respectively, by X-ray absorption near-edge structure (XANES) (Supplementary Fig. 21). Furthermore, sXRD studies on these designed samples at various states of charge (Supplementary Fig. 22) reveal Li_7/6_Ni_4/9_Ru_7/18_O_2_ (LR1) and Li_5/4_Ni_1/3_Ru_5/12_O_2_ (LR2) samples at charged state show similar isotropic structural evolution resembling that of Li_1.2_Ni_0.4_Ru_0.4_O_2_ sample. However, Li_4/3_Ni_2/9_Ru_4/9_O_2_ (LR3) sample does not exhibit a similar change; instead, the (003) peak splits to two peaks. In combination with Rietveld analysis, only 20.7% rocksalt phase in the final material is not sufficient to completely suppress the anisotropic structural change. Herein, *d*^3^ Ru^5+^ with partial flexibility in electronic structure can possibly accommodate both layered and rocksalt structure.

Alternatively, the utilization of Ru^5+^ (0.57 Å, 4*d*^3^) with smaller Ni^3+^ (0.56 Å) in Li_1.2_Ni_0.6_Ru_0.2_O_2_ or Ni^2+^ (0.69 Å) with Ru^4+^ (0.62 Å, 4*d*^4^) in Li_1.2_Ni_0.2_Ru_0.6_O_2_ leads to a layered structure (Fig. 6b). The formation of these layered oxides can be well explained by the ionic radius and electronic configuration of different cations. For example, in Li_1.2_Ni_0.6_Ru_0.2_O_2_, the small Ni^3+^ is the dominating cation and does not favor Li^+^ displacement to form rocksalt phase, whereas in Li_1.2_Ni_0.2_Ru_0.6_O_2_, the electronic configuration of dominating Ru^4+^ (4*d*^4^) plays a key role in the formation of the final phase. These results indicate that the considerations of TM ionic radius and electronic configuration are effective for the design of such layered-rocksalt intergrown materials. We note this design principle can be applied to abundant and low-cost 3*d* TMs, which is critically important for the further development of layered-rocksalt intergrown cathodes. One example is the design and synthesis of a series of compounds based on the combination of Ni^2+^, Fe^3+^, and Mn^4+^. Of these cations, Ni^2+^ (0.69 Å) and Fe^3+^ (0.645 Å) have similar ionic radius to Li^+^ (0.76 Å), facilitating cation mixing and rocksalt phase formation. In comparison, the role of Mn^4+^ (0.53 Å, 3*d*^3^) is similar to that of Ru^5+^ (0.57 Å, 4*d*^3^); its different ionic radius from Li^+^ and distortable electronic configuration enable the formation of a layered-rocksalt structure. As shown in Supplementary Fig. 23, the as-designed materials show evidence of the layered-rocksalt intergrown structure based on the relative ratio of (104) and (003) located at 44.7° and 19.0°, respectively. Furthermore, the HAADF-STEM image and EELS mapping are collected on one representative Li-Ni-Fe-Mn-O sample, Li_1.15_Ni_0.20_Fe_0.15_Mn_0.50_O_2_, showing the intergrown structure of the layered and rocksalt phases with uniform elemental distribution (Supplementary Fig. 24).

We would emphasize again that the layered-rocksalt intergrown material indeed inherits the advantages of both phases, displaying high-capacity and low-hysteresis electrochemical profiles with nearly zero-strain isotropic structural evolution upon electrochemical cycling. The intriguing finding of the coupled cationic TM “reduction” and anionic O oxidation with negligible irreversible oxygen release not only provides the practical optimism but also inspires future studies on the importance of TM-O interactions for oxygen activities in oxygen-redox-active systems. Overall, combination of these high-performance features in one single material is not trivial, making it a very promising direction for the search of advanced battery cathodes. Most importantly, layered and rocksalt phases are structurally compatible, so a large composition space is opened up for the search of commercially viable materials with such layered-rocksalt intergrown structure for advanced Li-ion batteries.

## Methods

### Synthesis

Li-rich metal oxides, Li_1.2_Ni_0.4_Ru_0.4_O_2_, along with other Li-Ni-Ru-O derivatives were prepared using Li_2_CO_3_, Ni(OH)_2_, and RuO_2_ as precursors. The precursors at designated stoichiometric amounts were first mixed on a Spex 8000 mill for 3 h, then fired at 450 °C for 3 h and 950 °C for 15 h in air, unless noted otherwise (Supplementary Fig. 25). The synthesis of this material via a solid-state reaction is very reproducible. Chemically delithiated samples were prepared by reacting Li_1.2_Ni_0.4_Ru_0.4_O_2_ with stoichiometric amounts of 0.1 M nitronium tetrafluoroborate (NO_2_BF_4_) in acetonitrile inside an Ar-filled glovebox (H_2_O < 0.1 p.p.m.) overnight. Onefold excess NO_2_BF_4_ was used to prepare fully delithiated sample. The final products were obtained by filtering and thoroughly washing the resulting mixtures by acetonitrile until the residual solution was clear, then drying under vacuum overnight. The compositions of final Li_*x*_Ni_0.4_Ru_0.4_O_2_ (1.2 ≤ *x* ≤ 0) and other Li-Ni-Ru-O derivatives were determined by inductively coupled plasma mass spectrometry analysis (Supplementary Table 7). Li-Ni-Fe-Mn-O derivatives were prepared by using Li_2_CO_3_, Ni(OH)_2_, FeC_2_O_4_, and MnCO_3_ as precursors. The precursors of designated stoichiometry were first mixed on a Spex 8000 mill for 3 h, followed by a calcination process at 450 °C for 3 h and 900 °C for 16 h in air.

### Characterization

sXRD was taken at the Advanced Photon Source at Argonne National Laboratory on beamline 11-BM. The beamline uses a sagittal focused X-ray beam with a high precision diffractometer circle and perfect Si (111) crystal analyzer detection for high sensitivity and resolution. In situ and ex situ sXRD was performed on beamline 11-3 equipped with a bent flat, side-scattering Si (311) monochromator at a fixed energy of 12,700 eV (*λ* = 0.976 Å) at Stanford Synchrotron Radiation Lightsource (SSRL). Powder XRD patterns were collected on a Bruker D2-Phaser with Cu Kα radiation (*λ* = 1.54178 Å). XRD patterns were analyzed by the conventional Rietveld method using the general structure analysis system package with the graphical user interface (EXPGUI). Scanning electron microscopy was performed on a JEOL JSM-7000F. High-resolution STEM images and electron diffraction patterns were obtained on JEM-2100F (JEOL) and HD2700C-dedicated STEM (Hitachi) with probe corrector at an accelerating voltage of 200 kV. HAADF-STEM images were filtered using Digital Micrograph software. Hard XAS measurements were performed on beamline 2-2 at SSRL in a transmission mode using a (220) monochromator. Higher harmonics in the X-ray beam was reduced by detuning the Si (220) monochromator. Energy was calibrated using the first inflection points in the spectra of Ni and Ru foil reference. XANES data were analyzed by SIXPACK software with the Photoelectron Energy Origin E_0_ determined by the first inflection point of the absorption edge jump. To visualize the valence state change of Ni at different states of charge, TXM was performed at beamline 6-2C at SSRL by loading the samples into quartz capillary tubes and sealing them inside a glovebox to avoid air exposure. A stack of transmission images (nominal spatial resolution of ~30 nm) recorded as the energy of the incoming X-rays was scanned across the Ni absorption *K*-edge. In the near-edge region, the energy step was set to be 1 eV for sufficient energy resolution. Over the pre-edge and post-edge regions, the energy was scanned at a larger step size of 10 eV, to cover a wide energy window for normalization of the X-ray absorption spectra. TXM data were analyzed using an in-house software package known as TXM-Wizard. RIXS maps were collected in the newly commissioned ultrahigh efficiency iRIXS endstation at Beamline 8.0.1 at the Advanced Light Sources. Sample surface was mounted 45° to the incident beam and the outgoing photon direction along the RIXS spectrograph is 90°. RIXS resolving power, other technical details, and data processing could be found in our previous report. All the cycled electrodes were immediately collected from the cells at designated voltages to minimize the side reactions between cycled electrodes and electrolyte, then vigorously washed by dimethyl carbonate solvent to remove the soluble surface species. All the dried electrodes were transferred into the experimental vacuum chamber through a specially designed sample transfer kit in an Ar-filled glovebox with no exposure to air. Operando DEMS measurements were taken on a customized Swagelok type cell connected to a high-pressure gas chromatography valve. The DEMS cells were initially rested at an open-circuit voltage for 6 h and the charge/discharge was done under potentiostatic control using a Bio-Logic SP-300 potentiostat.

### Electrochemistry

Electrodes were prepared from slurries containing 80 wt% of active material, 10 wt% of polyvinylidene fluoride binder, and 10 wt% acetylene carbon black (Denka, 50% compressed) in *N*-methylpyrrolidone solvent. The slurries were casted on carbon-coated aluminum current collectors (Exopack Advanced Coatings) using a doctor blade and then dried under vacuum at 120 °C overnight. Typical loading of the active materials was ~2.5 mg cm^−2^. 2032-type coin cells (Hohsen Corp.) containing Li metal, a Celgard 2400 separator, and 1 M LiPF_6_ electrolyte solutions in 1 : 2 w/w ethylene carbonate–diethyl carbonate (Daikin America) were assembled inside an Ar-filled glovebox (H_2_O < 0.1 p.p.m.). Galvanostatic charge and discharge were performed on a Maccor 4200 cycler at designated rates and voltages. A 1C current density was defined as 250 mA g^−1^.

## Supplementary information

Supplementary Information

## Data Availability

The data that support the findings of this study are available from the corresponding author upon reasonable request.
