# Supplementary material for: Layered-rocksalt intergrown cathode for high-capacity zero-strain battery operation
Source: Nat Commun. 2021 Apr 20;12:2348. doi: 10.1038/s41467-021-22527-z (PMC8058087; doi:10.1038/s41467-021-22527-z)
Supplement: Supplementary file 1 — Supplementary Information [file 41467_2021_22527_MOESM1_ESM.pdf]

**Supplementary Information for**

# Layered-Rocksalt Intergrown Cathode for High-Capacity Zero-Strain Battery Operation

Ning Li[1], Meiling Sun[1], Wang Hay Kan[2], Zengqing Zhuo[3], Sooyeon Hwang[4], Sara E. Renfrew[5], Maxim Avdeev[6], Ashfia Huq[7], Bryan D. McCloskey[1, 5], Dong Su[4], Wanli Yang[3*], Wei Tong[1*]

[1]*Energy Storage and Distributed Resources Division, Lawrence Berkeley National Laboratory, Berkeley, CA 94720 USA*

[2]*Dongguan Neutron Science Center, Dongguan, Guangdong, 523803 China*

[3]*Advanced Light Source, Lawrence Berkeley National Laboratory, Berkeley, CA 94720 USA*

[4]*Center for Functional Nanomaterials, Brookhaven National Laboratory, Upton, NY 11973 USA*

[5]*Department of Chemical and Biomolecular Engineering, University of California, Berkeley, CA 94720, USA*

[6]*Australian Nuclear Science and Technology Organisation (ANSTO), Lucas Heights, New South Wales 2234, Australia*

[7]*Neutron Scattering Science Directorate, Oak Ridge National Laboratory, Oak Ridge, Tennessee 37831, United States*

[*]Correspondence: wlyang@lbl.gov, weitong@lbl.gov

**Supplementary Note 1.**

The Rietveld refinement of the ND reflections of chemically delithiated $Li_{0.5}Ni_{0.4}Ru_{0.4}O_2$ indicates the preference of $Li^+$ extraction from rocksalt rather than layered phase during initial $Li^+$ extraction. A specific constrain is created in the refinement process as described below:

Firstly, a routine two-phase refinement process was conducted, including the optimization of parameters for cell parameter, thermal parameter, atomic position for O in layered phase, zero position, phase-fraction, scale, profile, and background. At this stage, the molar ratio between lithium and the transition metal ions were assumed to be the same in both layered and rock-salt phases. A weighted residue value (wRp) of 3.95% was obtained, indicating that the simulated and experimental patterns were in pretty good agreement. Then, a new constrain was created to allow a movement of lithium ions from the layered to rock-salt phase but their total quantity was kept constant. Based on our previous refinement analysis for the pristine phase that the molar ratio between layered and rock-salt was found to be 70:30, the constrain was set such as the change of the quantity of lithium ions in 3a in the layered phase (3a is the Li site; 3b is the TM site in conventional $LiMO_2$ of $R\bar{3}m$) times 6 was equal to the change of quantity of lithium ions in 4a (4a is the cation site; 4b is the O site) in the rock-salt phase times 7. Once this constrain was set, the occupancy parameters for the lithium ion in 3a position in the layered phase and the 4a position in the rock-salt phase were allowed to refine. Surprisingly, the weighted residue value was further reduced to 3.62%. The summary of the result indicates that lithium content was lower in the rock-salt phase than that in the layered phase.

To verify this result, two refinement scenarios were compared. In one case, we arbitrarily removed the lithium ion from the layered phase first, by maintaining the nominal composition of rock-salt same as $Li_{0.5}Ni_{0.4}Ru_{0.4}O_2$. The slightly large weighted residue value of 4.2% was obtained.

In a second case, we arbitrarily remove the lithium-ion from the rock-salt phase first, by maintaining the nominal composition of layered phase as $Li_{0.5}Ni_{0.4}Ru_{0.4}O_2$. A weighted residue of 3.63% was obtained. The results suggest that lithium ions have a preferential removal mechanism from the rock-salt phase than layered phase.

The chemical composition of $Li_{0.5}Ni_{0.4}Ru_{0.4}O_2$ is determined by inductively coupled plasma mass spectrometry (ICP-MS) analysis. Assuming full delithiation of rocksalt phase (30 mol%), the chemical composition of the layered component in $Li_{0.5}Ni_{0.4}Ru_{0.4}O_2$ is $Li_{0.714}Ni_{0.4}Ru_{0.4}O_2$.

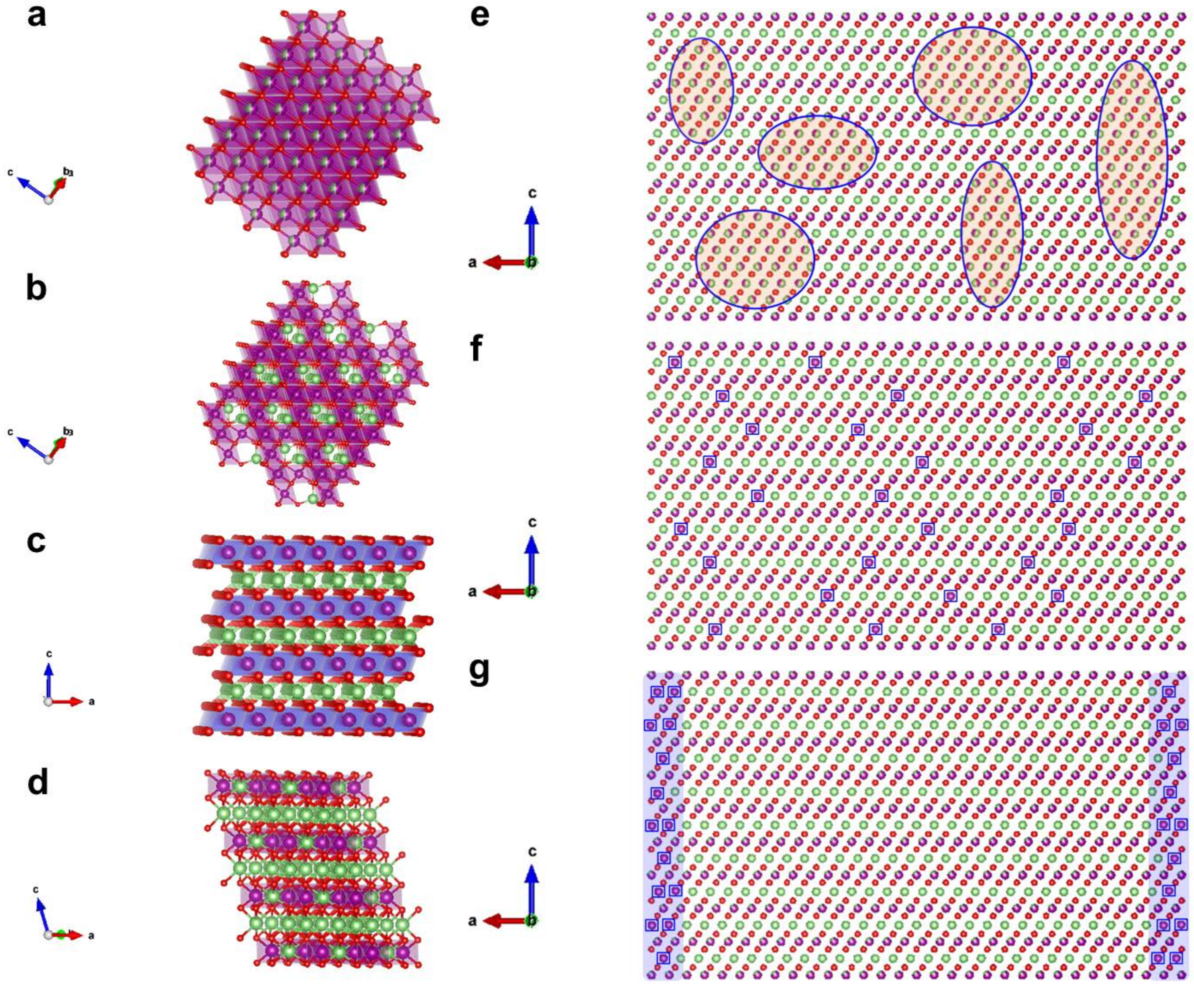


**Supplementary Fig. 1 | Crystal structure of common lithium metal oxides observed in Li-ion cathode.** **(a)** Cubic rocksalt oxide of $Fm\overline{3}m$; (**b**) cubic spinel oxide of $F3dm$; (**c**) hexagonal layered oxide of $R\overline{3}m$; (**d**) monoclinic Li-rich layered oxide of $C/2m$; and three scenarios in layered oxide cathode including (**e**) intergrown layered-rocksalt phase proposed in present work, the rocksalt component in the as-proposed layered-rocksalt intergrown oxide is electrochemically active and the sole layered component can't account for such a high degree of $Li^+$ extraction/insertion and the intergrown phase exhibits an isotropic lattice change upon cycling.; (**f**) layered phase with Li/Ni intermixing, Li/Ni intermixing in the layered cathode can alleviate the phase transition, to some extent, but it can't suppress its intrinsic anisotropic lattice change; and (**g**) layered phase with surface rocksalt, the rocksalt phase that presents a thin layer (a couple nanometer) at the surface during synthesis or upon cycling is electrochemically inactive densified layer with low electronic and ionic conductivity. The green, purple, and red spheres represented Li, TM, and O, respectively.

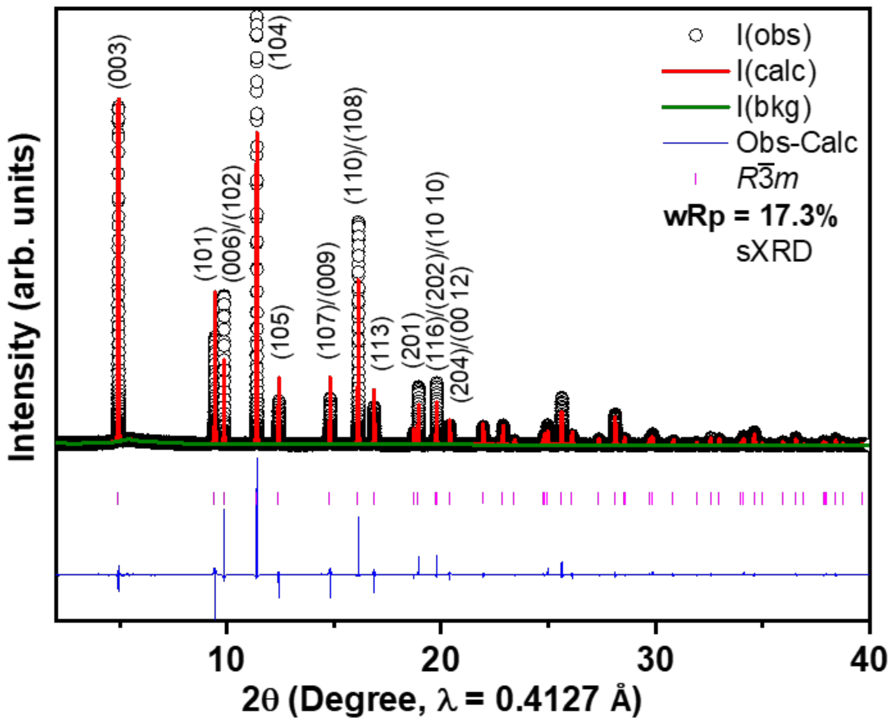


**Supplementary Fig. 2 | Rietveld refinement of sXRD of pristine $Li_{1.2}Ni_{0.4}Ru_{0.4}O_2$ based on $R\bar{3}m$ space group with no Li/Ni intermixing.** Lattice parameters are $a$ = 2.94062(1) Å, $c$ = 14.40222(5) Å and $V$ = 107.85(5) Å$^3$, with a R-factor of 17.3%. Refinement based on $R\bar{3}m$ space group leads to a good fit in the peak position, but a discrepancy in the intensity ratio of reflection (003)/(104), located around 4.9 and 11.4°, respectively, is observed with a R-factor of 17.3%, indicating the possibility of Li/Ni mixing or a secondary rocksalt phase.

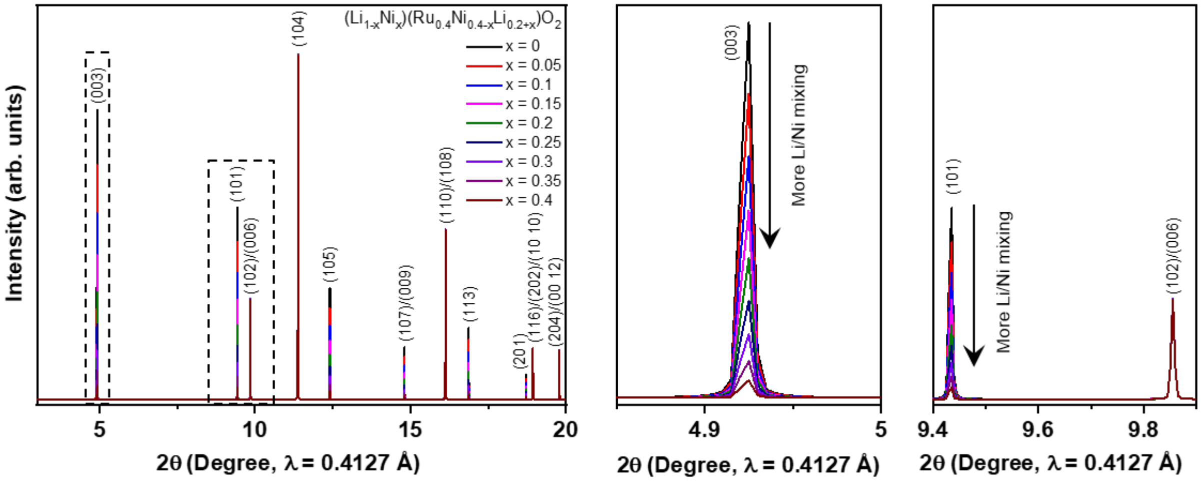


**Supplementary Fig. 3 | Simulated XRD patterns for $Li_{1.2-z}Ni_z[Li_{0.2+z}Ni_{0.4-z}Ru_{0.4}]O_2$ based on $R\bar{3}m$ space group with Li/Ni intermixing (z value).** XRD patterns are simulated based on $R\bar{3}m$ space group with Li/Ni mixing. General formula for Rietveld refinement is $Li_{1-z}Ni_z[Li_{0.2+z}Ni_{0.4-z}Ru_{0.4}]O_2$ ($z = 0 – 0.2$). The intensity of (hkl) reflections, e.g., (003) and (101), with respect to (104) varies with the degree of Li/Ni mixing (z value).

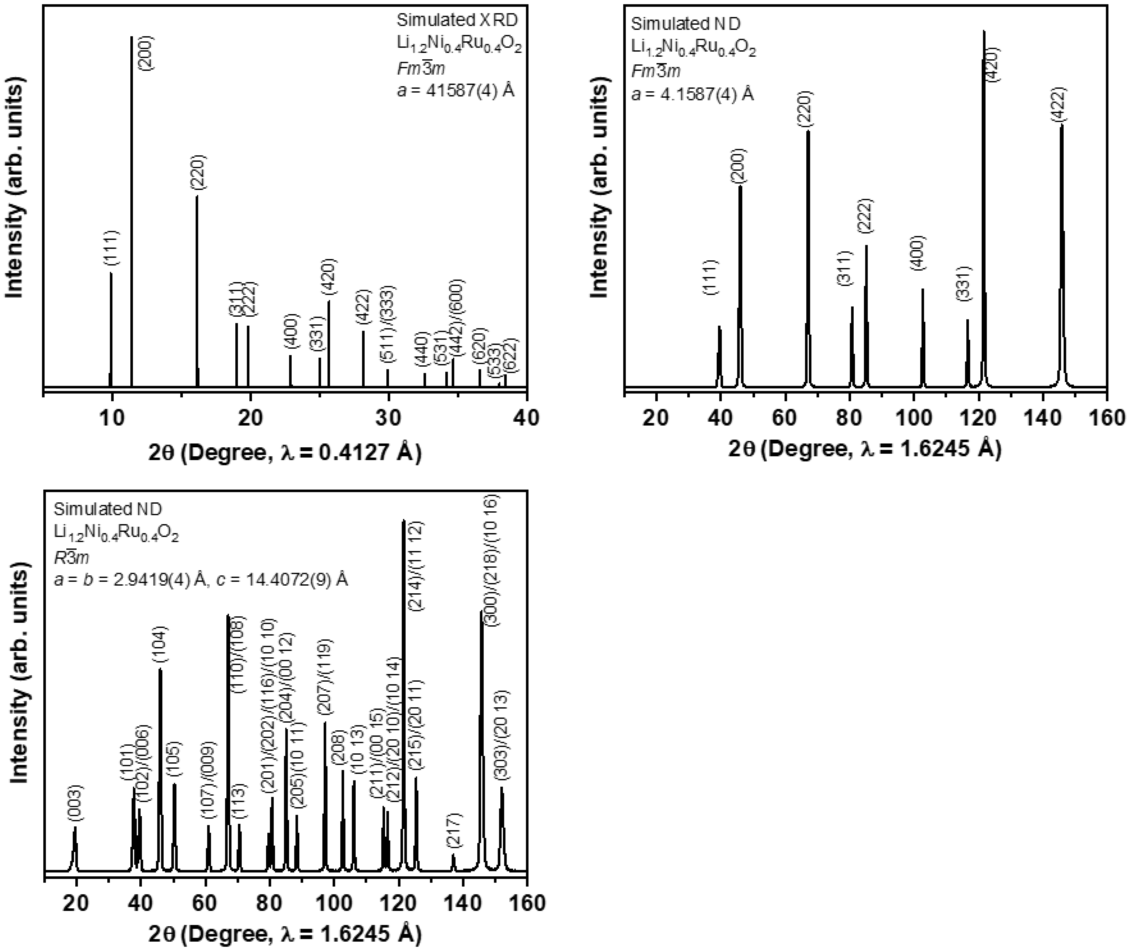


**Supplementary Fig. 4 | Simulated XRD and NR patterns for $Li_{1.2}Ni_{0.4}Ru_{0.4}O_2$ based on $R\bar{3}m$ and $Fm\bar{3}m$ space groups.**

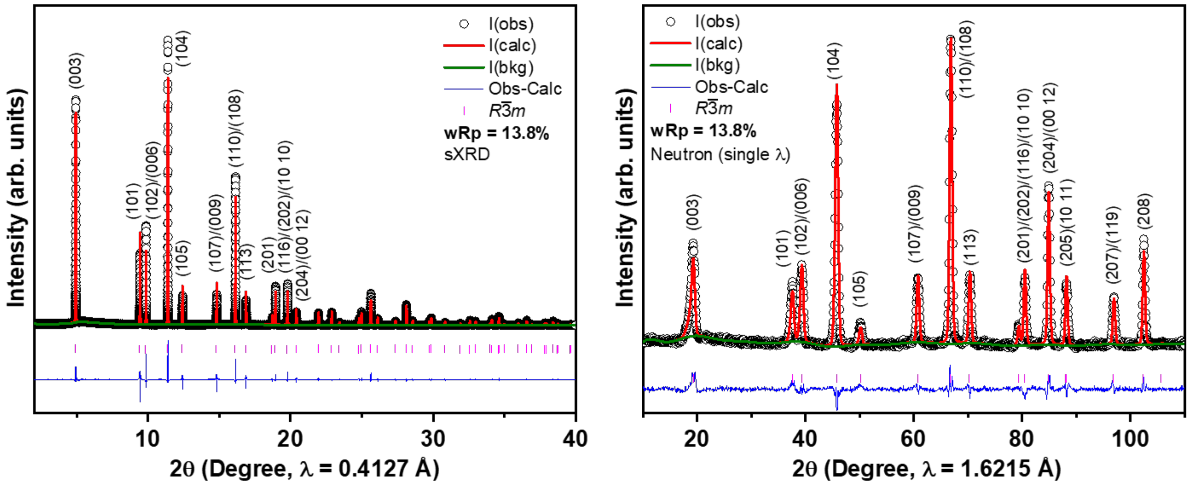


**Supplementary Fig. 5 | Joint refinement of sXRD and ND for $Li_{1.12}Ni_{0.08}[Li_{0.28}Ni_{0.32}Ru_{0.4}]O_2$ based on $R\overline{3}m$ single phase model with Li/Ni intermixing.** A final R-factor of 13.8% is obtained from the joint refinement along with ~8% Li/Ni mixing. Refinement based on $R\overline{3}m$ space group with Li/Ni intermixing leads to a better fit with a final R-factor of 13.8%. Lattice parameters are $a = b = 2.94044(1)$ Å, $c = 14.40143(3)$ Å, $\alpha = \beta = 90°$, $\gamma = 120°$ and $V = 107.835(2)$ Å$^3$ and ~8.0% Li/Ni mixing.

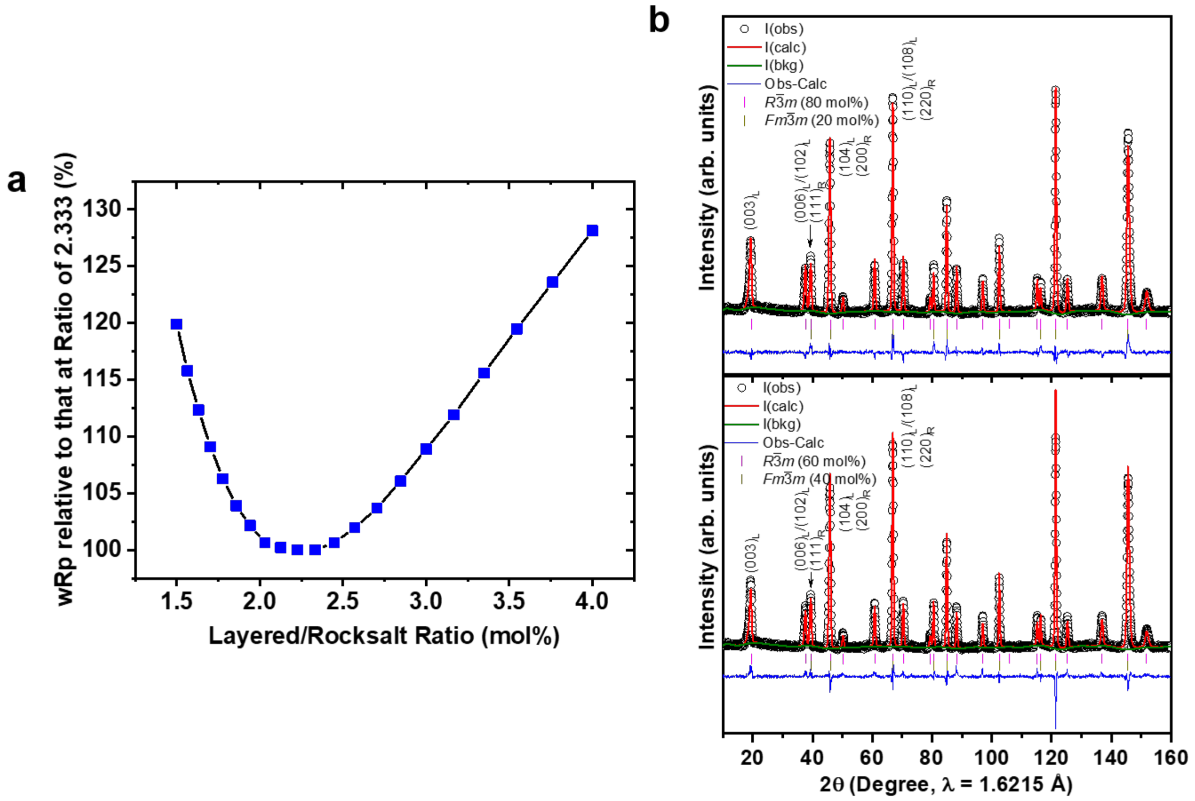


**Supplementary Fig. 6 | Rietveld analysis at various layered/rocksalt ratios.** (**a**) Percentage of wRp at the molar ratio from 1.5 to 4.0 relative to 2.333, showing the layered/rocksalt ratio of 2.333 (70: 30) gives the best fit. (**b**) Rietveld refinement results at the layered/rocksalt ratio of 4.0 (80: 20) and 1.5 (60: 40). Critical reflections are indexed as $(hkl)_L$ and $(hkl)_R$ for $R\bar{3}m$ and $Fm\bar{3}m$, respectively.

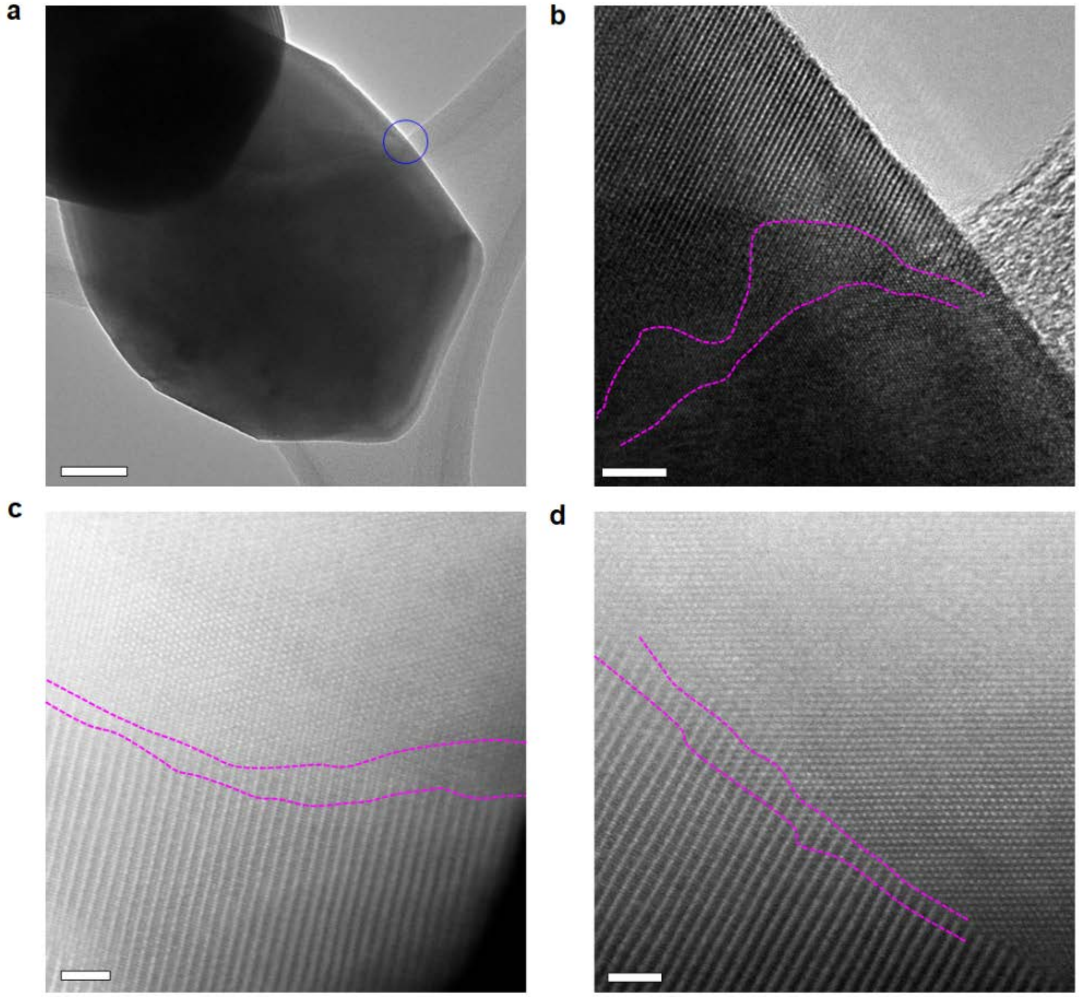


**Supplementary Fig. 7 | HRTEM and STEM images of pristine $Li_{1.2}Ni_{0.4}Ru_{0.4}O_2$.** (**a**, **b**) HRTEM images with a scale bar of 50 and 5 nm, respectively. (**c**, **d**) STEM images with a scale bar of 2 nm, showing layered-rocksalt intergrown structure. Pink lines highlight the structurally compatible region with varied TM distribution in the Li slabs.

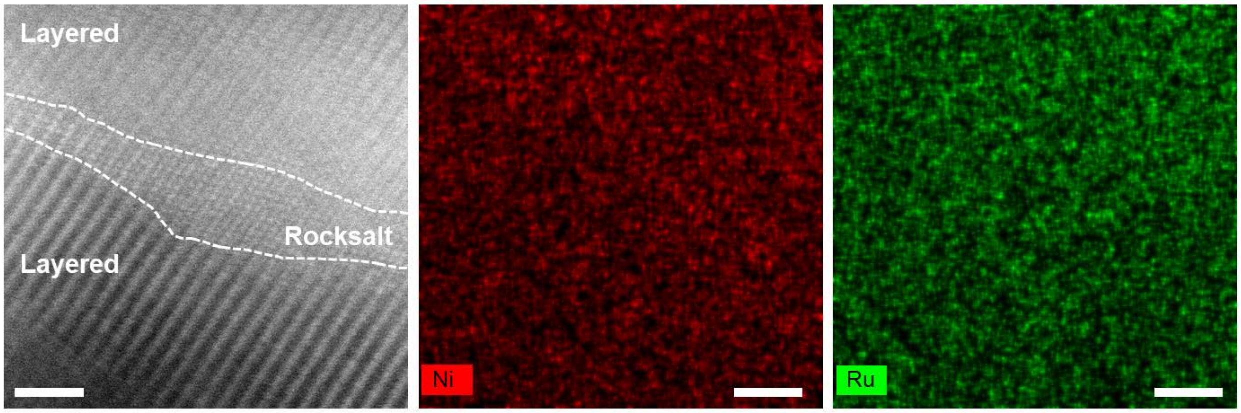


**Supplementary Fig. 8 | HADDF-STEM image and EELS mapping of pristine $Li_{1.2}Ni_{0.4}Ru_{0.4}O_2$.** HADDF-STEM image clearly shows both layered and rocksalt regions. EELS mapping shows the homogenous elemental distribution in both regions. Elemental mapping from EELS measurement indicates the TMs are quite consistent, showing no great change in TM ratio, in the layered and rocksalt regions. Scale bar is 2 nm.

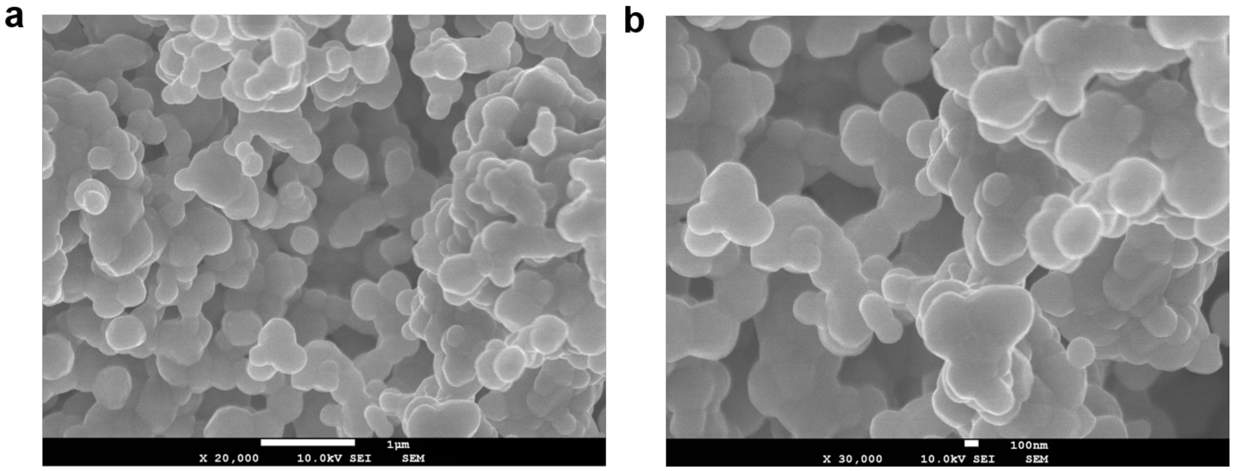


**Supplementary Fig. 9 | Morphology of pristine $Li_{1.2}Ni_{0.4}Ru_{0.4}O_2$.** Scale bar in (a) and (b) is 1 μm and 100 nm, respectively. The particle size of the as-produced $Li_{1.2}Ni_{0.4}Ru_{0.4}O_2$ is ~500 nm, which is directly used for electrochemical characterization without further nanoscaling.

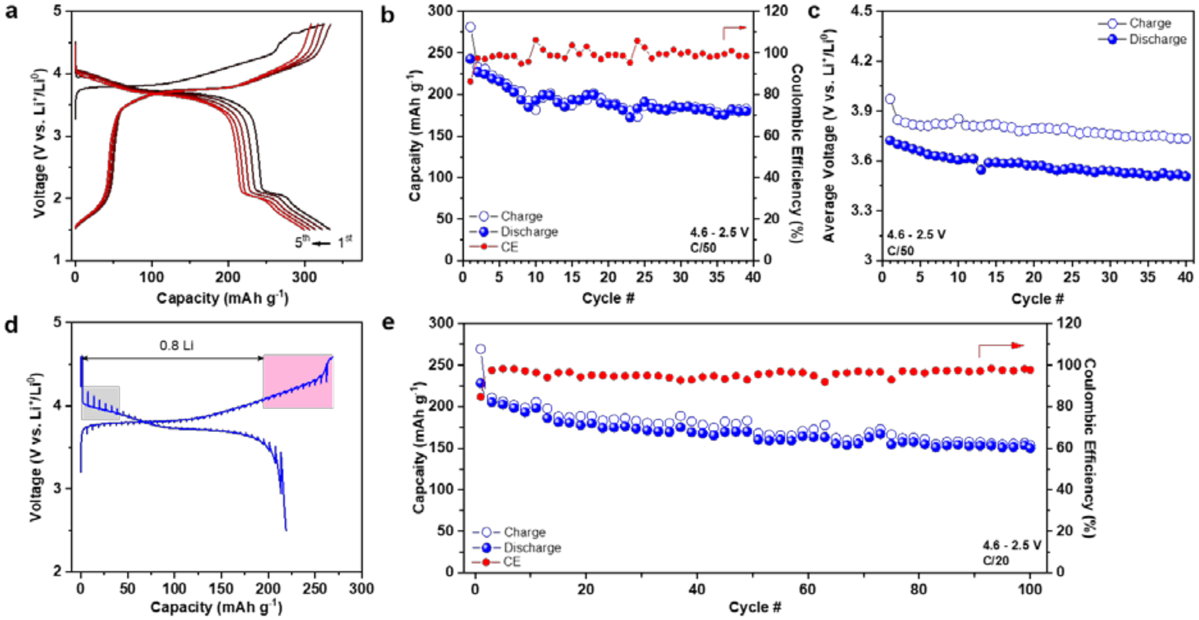


**Supplementary Fig. 10 | Electrochemical properties of $Li_{1.2}Ni_{0.4}Ru_{0.4}O_2$.** (**a**) Voltage profiles during the first five cycles between 4.8 and 1.5 V. (**b**) Cycling performance and (**c**) average voltage between 4.6 and 2.5 V at C/50, showing a slight voltage decay upon cycling. (**d**) GITT test showing the kinetics of various redox during charge/discharge between 4.6 and 2.5 V. Pink area indicates additional capacity beyond theoretical TM redox, grey area highlights slightly high polarization region during discharge. (**e**) Cycling performance between 4.6 and 2.5 V at C/20.

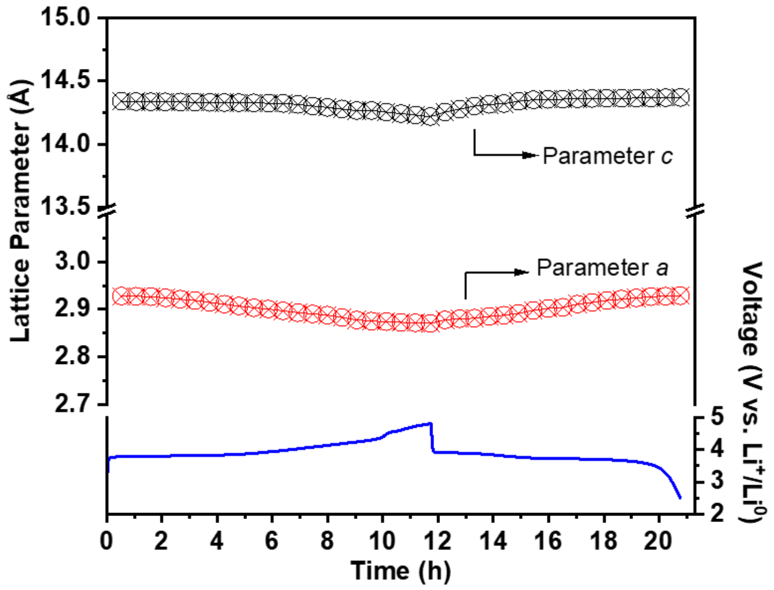


**Supplementary Fig. 11 | Lattice parameter changes upon electrochemical cycling.** Lattice parameters are obtained by Rietveld refinement of the in situ XRD patterns based on $R\bar{3}m$ through Le Bail fit, showing a slight change in lattice *c* (~1%) upon charging and discharging.

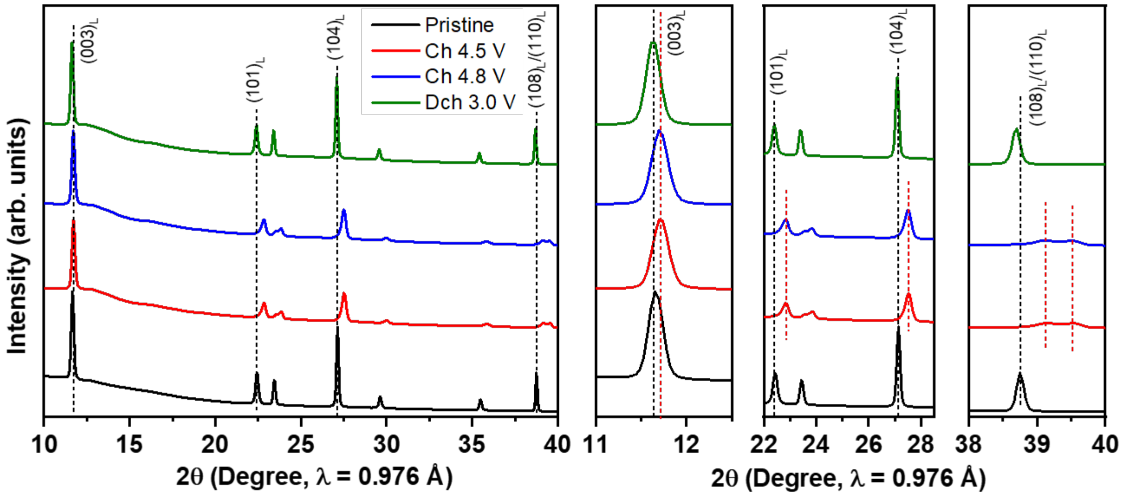


**Supplementary Fig. 12 | *Ex situ* sXRD of $Li_{1.2}Ni_{0.4}Ru_{0.4}O_2$ electrode at different states of charge.** Black and red dash lines mark the characteristic reflections of the layered phase at pristine and charged state, respectively.

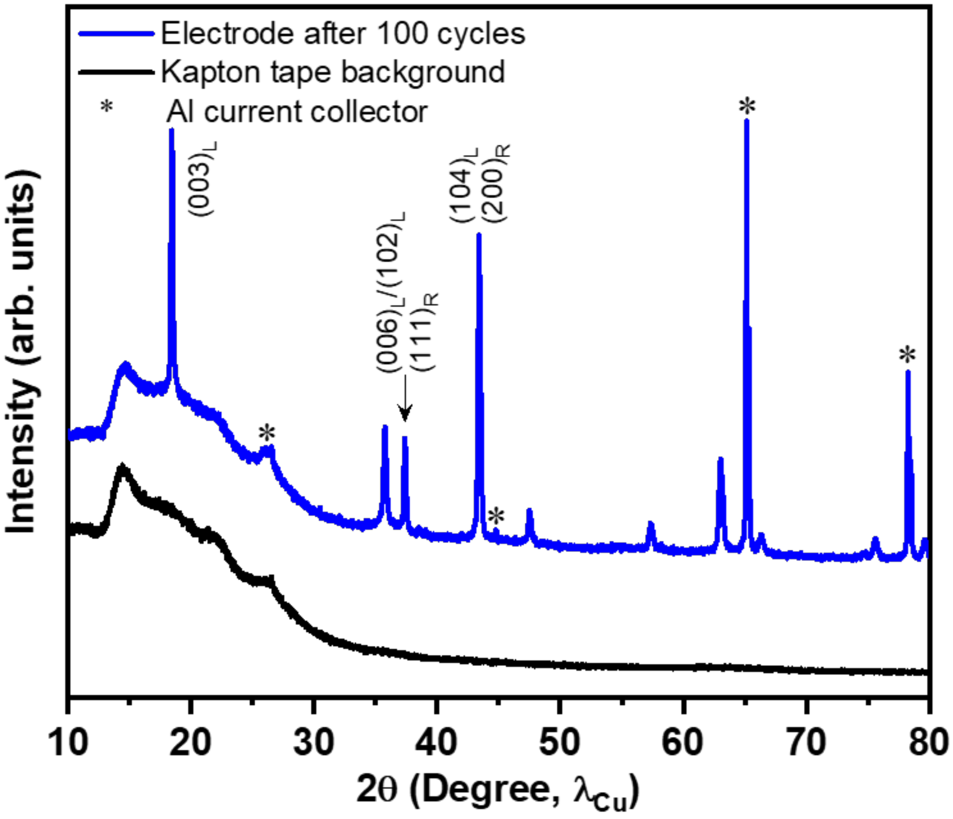


**Supplementary Fig. 13 | XRD pattern of $Li_{1.2}Ni_{0.4}Ru_{0.4}O_2$ electrode after 100 cycles at C/20.** The XRD pattern shows the well-crystallized electrode after 100 cycles. All the major peaks remain and the intensity ratio of $(003)_L/(104)_L$, located at 18.5 and 43.4º, also remains after 100 cycles in comparison with those at the pristine state, indicating the robust layered-rocksalt intergrown structure upon long-term cycling. Critical reflections are indexed as $(hkl)_L$ and $(hkl)_R$ for $R\bar{3}m$ and $Fm\bar{3}m$, respectively.

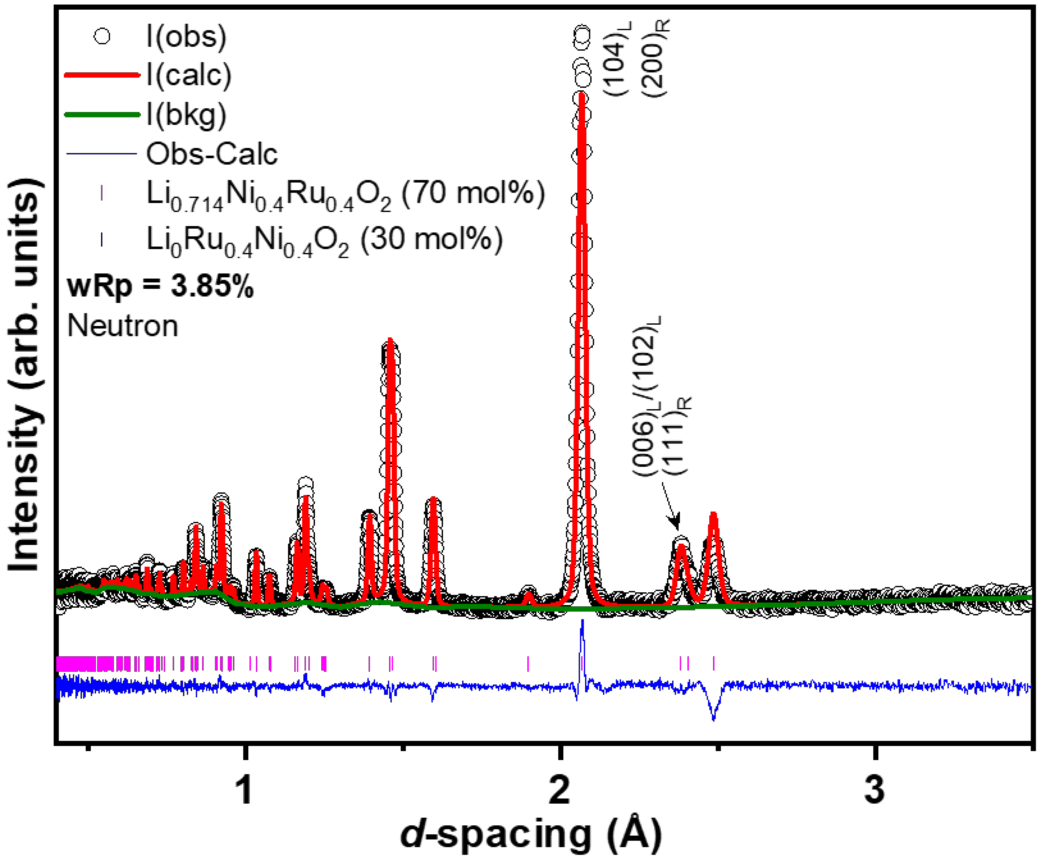


**Supplementary Fig. 14 | Refinement of TOF-ND for $Li_{0.5}Ni_{0.4}Ru_{0.4}O_2$ based on $R\bar{3}m$ and $Fm\bar{3}m$ biphasic phase model.** Rietveld refinement with a final R-factor of 3.9% suggests $Li^+$ is initially extracted from the rocksalt phase (only 0.36 $Li^+$ for 30% of total 1.2 $Li^+$), and then from layered phase (another 0.34 $Li^+$ for 0.7 $Li^+$ extraction), resulting in 70% layered $Li_{0.714}Ni_{0.4}Ru_{0.4}O_2$ and 30% $Li_0Ni_{0.4}Ru_{0.4}O_2$ rocksalt. Only critical reflections for $R\bar{3}m$ and $Fm\bar{3}m$ are indexed as $(hkl)_L$ and $(hkl)_R$, respectively.

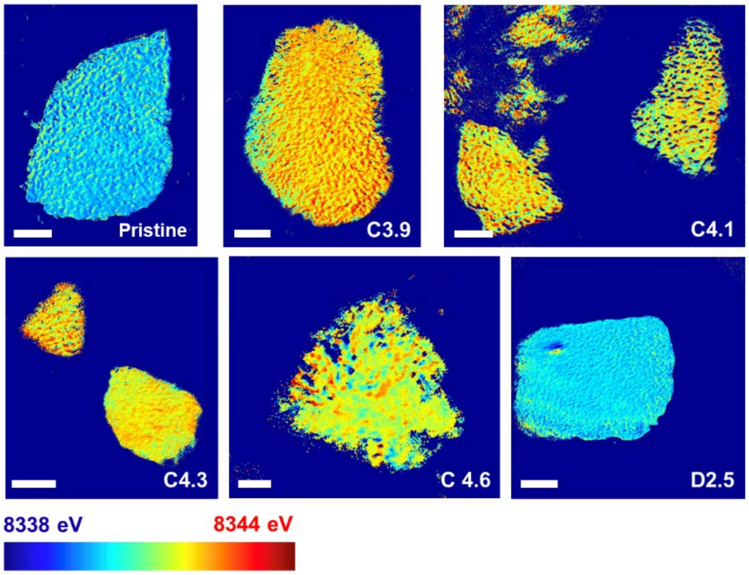


**Supplementary Fig. 15 | 2D TXM images of $Li_{1.2}Ni_{0.4}Ru_{0.4}O_2$ at different states of charge.** Scale bar is 5 μm. The 2D TXM images clearly demonstrate the valence state of Ni initially increases and then slightly decreases during charge, which is consistent with previously proposed reductive coupling effect.

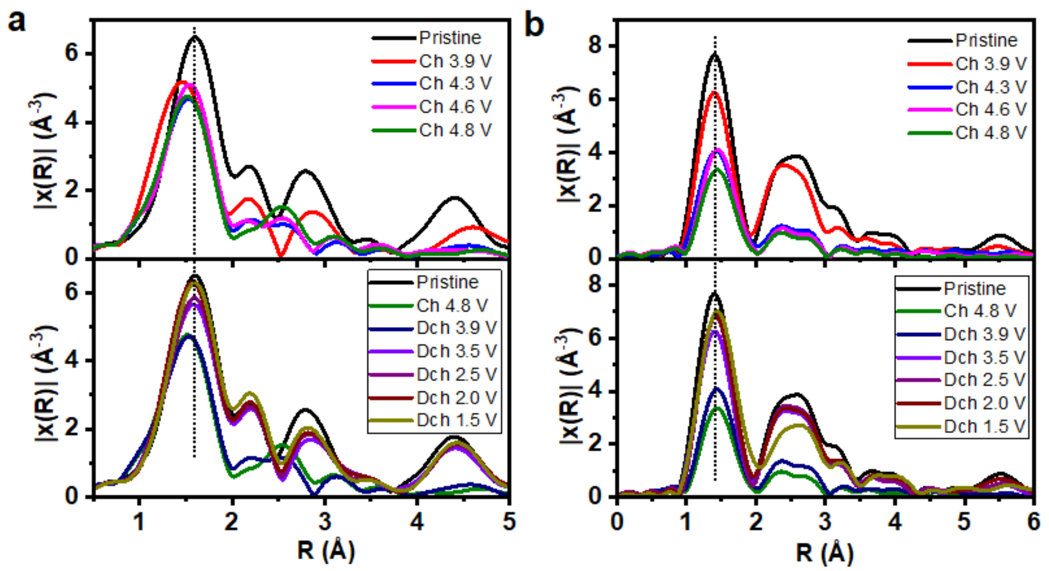


**Supplementary Fig. 16 | EXAFS of TM at different electrochemical states.** (**a**) Ni and (**b**) Ru. Top and bottom panels show charged and discharged states, respectively.

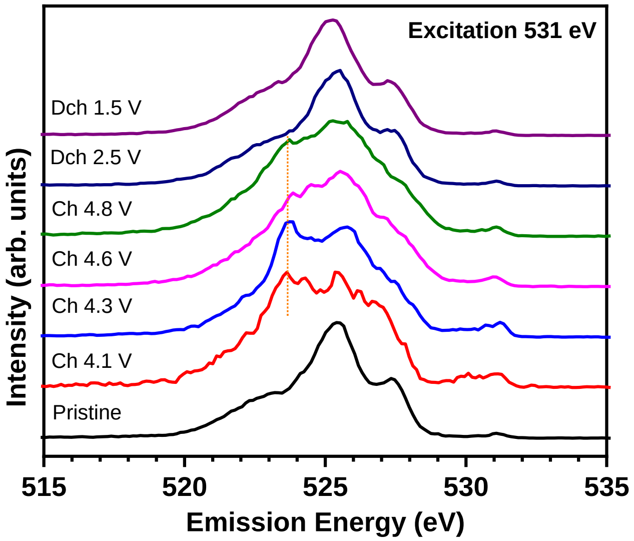


**Supplementary Fig. 17 | O *K*-edge RIXS spectral cuts of $Li_{1.2}Ni_{0.4}Ru_{0.4}O_2$ at different electrochemical states.** RIXS spectra are obtained by plotting the photon intensity upon emission energy with the excitation energy of 531 eV, showing the enhanced 523.7 eV feature is clear at high voltage states and disappears at the discharged states, revealing the reversible lattice oxygen redox reactions. Broader RIXS features observed at the high voltage states are typical for the highly oxidized systems, because high oxidization states correspond to the strong TM-O hybridization.

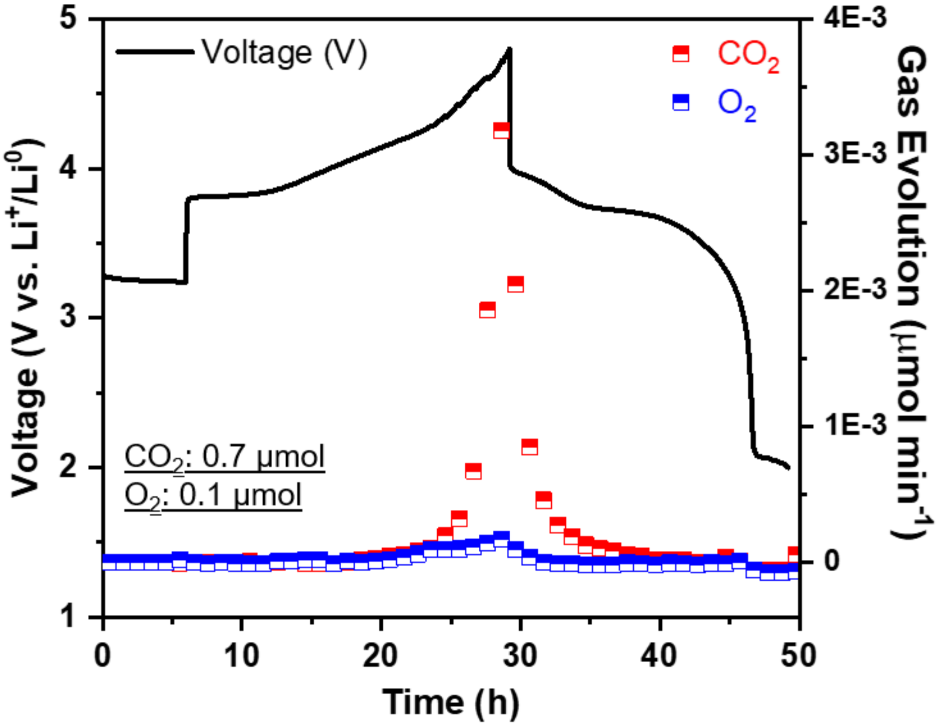


**Supplementary Fig. 18 | Gas evolution of Li1.2Ni0.4Ru0.4O2 determined by DEMS.** $CO_2$ evolution bursts after 4.3 V charge with minimal $O_2$ detected during the first cycle. Total active Li1.2Ni0.4Ru0.4O2 is 12.22 mg. Gas evolution rate of $O_2$ and $CO_2$ is 0.053 and 0.005 μmol/mg, respectively.

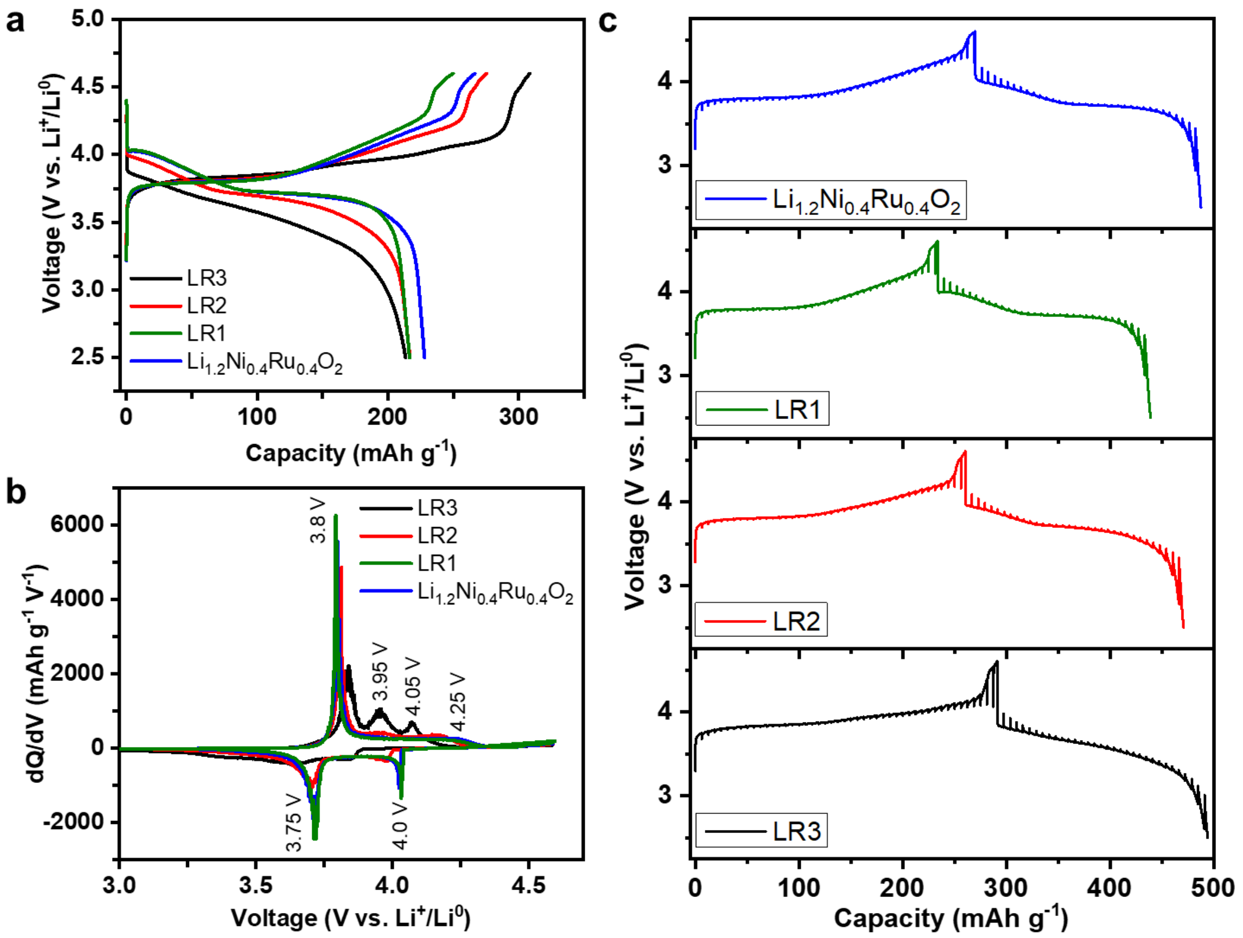


**Supplementary Fig. 19 | Electrochemical properties of new layered-rocksalt intergrown oxides based on $Ni^{2+}/Ru^{5+}$ combination.** Samples of $Li_{7/6}Ni_{4/9}Ru_{7/18}O_2$, $Li_{5/4}Ni_{1/3}Ru_{5/12}O_2$, and $Li_{4/3}Ni_{2/9}Ru_{4/9}O_2$ are labelled as LR1, LR2, and LR3, respectively. (**a**) The first voltage profiles. (**b**) dQ/dV plots. (**c**) GITT test. Cells were cycled between 4.6 and 2.5 V.

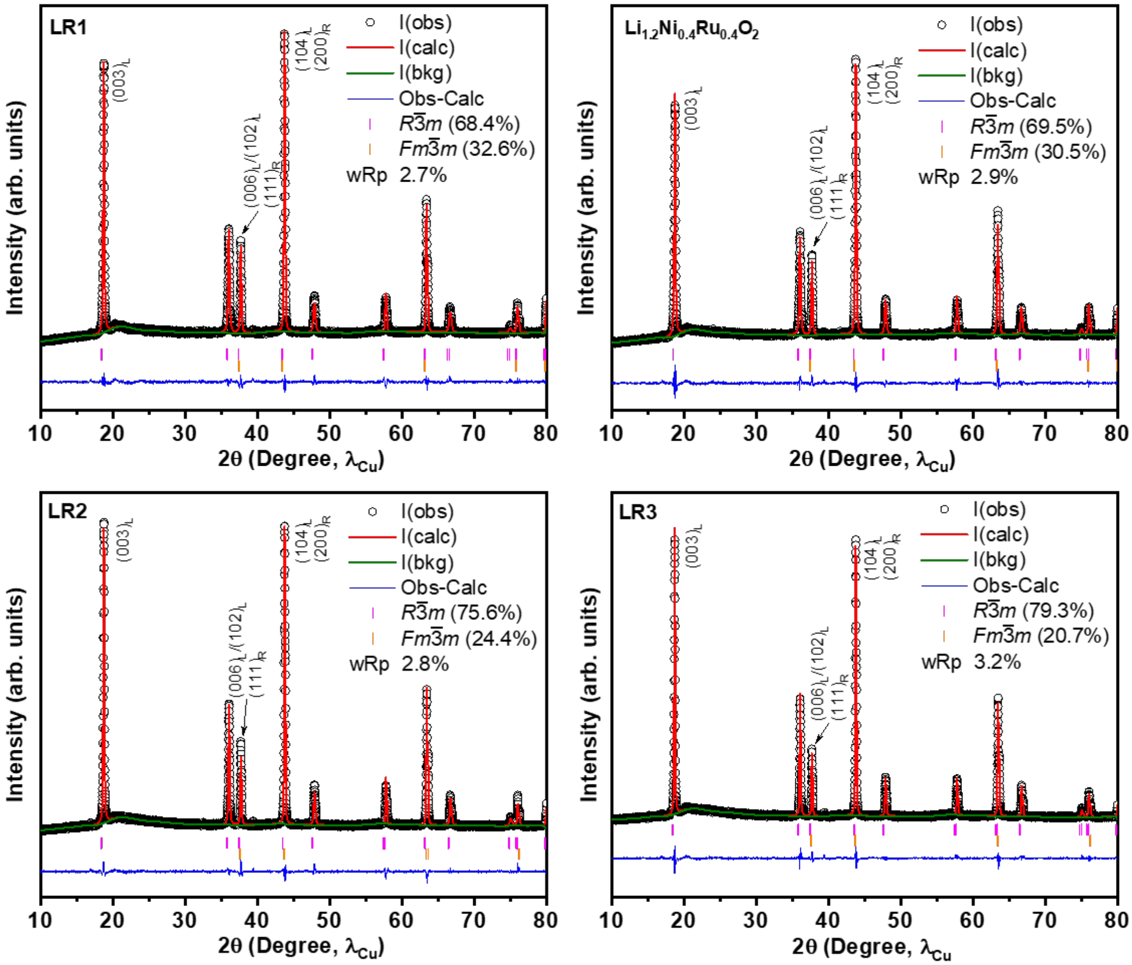


**Supplementary Fig. 20 | XRD refinement analysis of $Li_{1-a-b}Ni^{2+}_aRu^{5+}_bO_2$ samples based on $Ni^{2+}/Ru^{5+}$ combination.** Samples of $Li_{7/6}Ni_{4/9}Ru_{7/18}O_2$, $Li_{5/4}Ni_{1/3}Ru_{5/12}O_2$, and $Li_{4/3}Ni_{2/9}Ru_{4/9}O_2$ are labelled as LR1, LR2, and LR3, respectively. They exhibit the layered-rocksalt intergrown structure with rocksalt component gradually increasing with Ni content. Only critical reflections for $R\bar{3}m$ and $Fm\bar{3}m$ are indexed as $(hkl)_L$ and $(hkl)_R$, respectively.

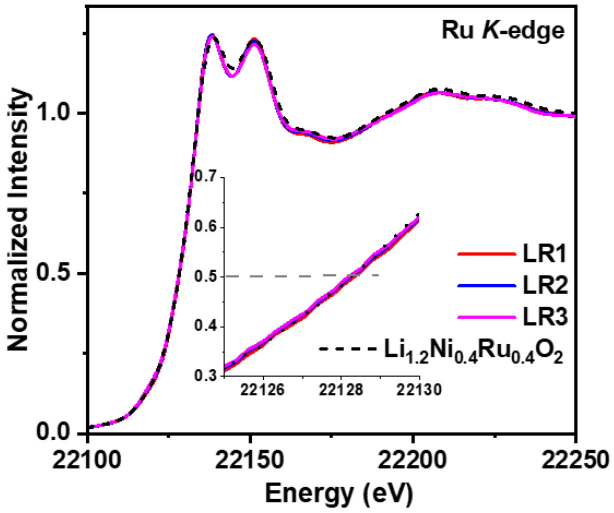


**Supplementary Fig. 21 | XANES Ru and Ni *K*-edge of all samples based on $Ni^{2+}/Ru^{5+}$ combination.** Samples of $Li_{7/6}Ni_{4/9}Ru_{7/18}O_2$, $Li_{5/4}Ni_{1/3}Ru_{5/12}O_2$, and $Li_{4/3}Ni_{2/9}Ru_{4/9}O_2$ are labelled as LR1, LR2, and LR3, respectively. Ni and Ru in all samples are 2+ and 5+, respectively, well consistent with the proposed design principle.

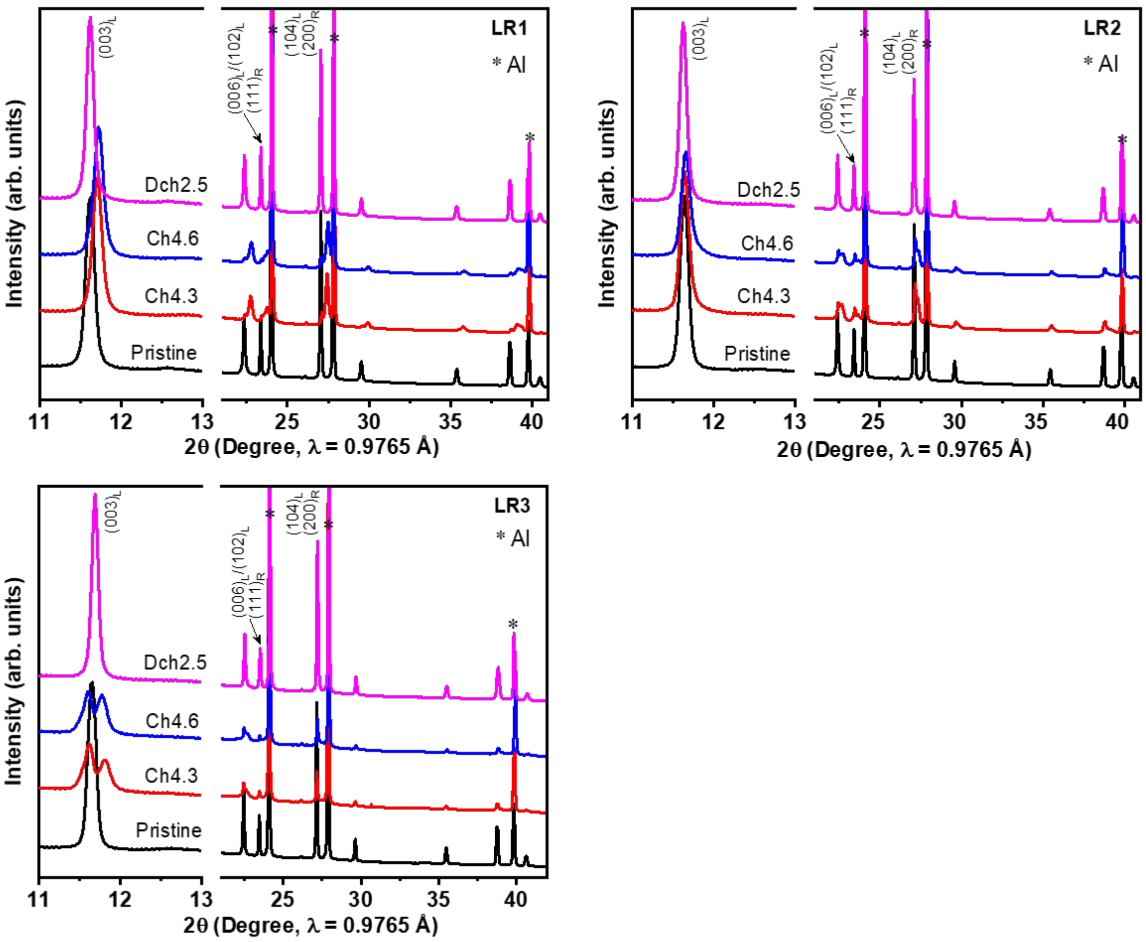


**Supplementary Fig. 22 | Phase evolution of designed samples based on $Ni^{2+}/Ru^{5+}$ combination.** Samples of $Li_{7/6}Ni_{4/9}Ru_{7/18}O_2$, $Li_{5/4}Ni_{1/3}Ru_{5/12}O_2$, and $Li_{4/3}Ni_{2/9}Ru_{4/9}O_2$ are labelled as LR1, LR2, and LR3, respectively. LR1 and LR2 exhibit similar isotropic structural evolution, however, LR3 does not follow the same trend due to the lower content of rocksalt. Only critical reflections for $R\bar{3}m$ and $Fm\bar{3}m$ are indexed as $(hkl)_L$ and $(hkl)_R$, respectively.

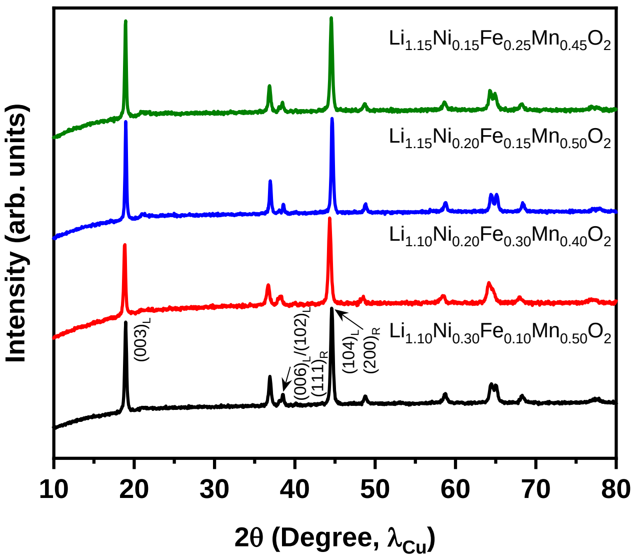


**Supplementary Fig. 23 |** XRD patterns of new Li-rich metal oxides of Ni/Fe/Mn combination. Li-Ni-Fe-Mn-O compounds of $Ni^{2+}/Fe^{3+}/Mn^{4+}$ combination show the evidence of the layered-rocksalt intergrown structure based on the relative ratio of $(104)_L$ and $(003)_L$ reflections located at 44.7 and 19.0°, respectively. Only critical reflections for $R\bar{3}m$ and $Fm\bar{3}m$ are indexed as $(hkl)_L$ and $(hkl)_R$, respectively.

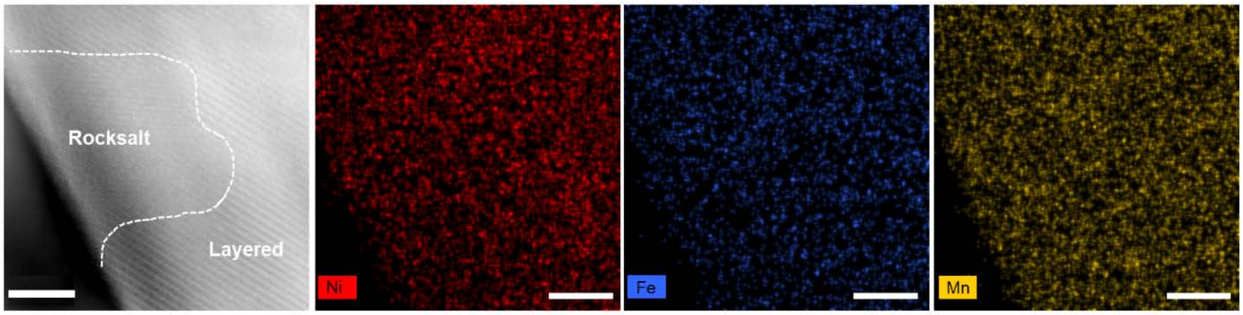


**Supplementary Fig. 24 | HADDF-STEM image and EELS mapping of pristine $Li_{1.15}Ni_{0.20}Fe_{0.15}Mn_{0.50}O_2$.** HADDF-STEM image shows the layered and rocksalt regions. EELS mapping shows the homogenous elemental distribution in both regions. Scale bar is 4 nm.

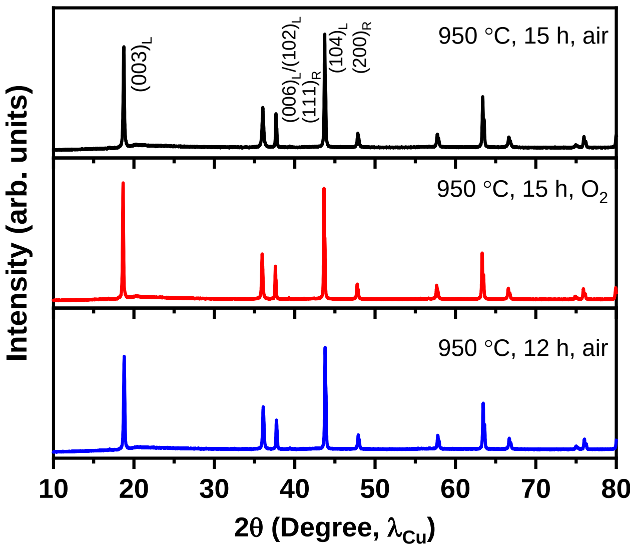


**Supplementary Fig. 25 | Synthesis of $Li_{1.2}Ni_{0.4}Ru_{0.4}O_2$ at different conditions.** The obtained XRD patterns show the effects of annealing conditions (temperature, time, and $O_2$ atmosphere) on the phase formation. Synthesis under $O_2$ flow, annealing temperature and time. The potential influence of oxidizing atmosphere is the oxidation state of Ni, as a result of $Ni^{2+}$ and $Ni^{3+}$ (if any) with different ionic radius, which would affect the cation mixing and phase formation (layered vs. rocksalt). The relative intensity of $(003)_L/(104)_L$ reflection at 18.5 and 43.4°, respectively, suggests a lower content for rocksalt phase under $O_2$. Meanwhile, a shorter annealing time does not lead to an obvious change based on XRD analysis.

**Supplementary Table 1.** Refined structural parameters of pristine $Li_{1.2}Ni_{0.4}Ru_{0.4}O_2$ based on biphasic $R\bar{3}m$ and $Fm\bar{3}m$ model.

| Pristine $Li_{1.2}Ni_{0.4}Ru_{0.4}O_2$: $R\bar{3}m$ | | | | | | |
|---|---|---|---|---|---|---|
| Atom | Wyckoff | x | y | z | Occupancy | Uiso |
| Li(1) | 3 | 0 | 0 | 0.50000 | 1.0000 | 0.0341(7) |
| Li(2) | 3 | 0 | 0 | 0 | 0.2000 | 0.0035(5) |
| Ru(1) | 3 | 0 | 0 | 0 | 0.4000 | 0.0035(5) |
| Ni(1) | 3 | 0 | 0 | 0 | 0.4000 | 0.0035(5) |
| O(1) | 6 | 0 | 0 | 0.25716(4) | 1.0000 | 0.0078(2) |
| Pristine $Li_{1.2}Ni_{0.4}Ru_{0.4}O_2$: $Fm\bar{3}m$ | | | | | | |
| Li(1) | 4 | 0 | 0 | 0 | 0.6000 | 0.0034(1) |
| Ni(1) | 4 | 0 | 0 | 0 | 0.2000 | 0.0034(1) |
| Ru(1) | 4 | 0 | 0 | 0 | 0.2000 | 0.0034(1) |
| O(1) | 4 | 0 | 0 | 0.5000 | 1.0000 | 0.0091(3) |

Pristine $Li_{1.2}Ni_{0.4}Ru_{0.4}O_2$ lattice parameter: $R\bar{3}m$, 70 mol%, $a = b = 2.94194(7)$ Å, $c = 14.40729(2)$ Å, $\alpha = \beta = 90^o$, $\gamma = 120^o$, $V = 107.990(1)$ Å$^3$; $Fm\bar{3}m$, 30 mol%, $a = b = c = 4.15874(6)$ Å, $\alpha = \beta = \gamma = 90^o$, $V = 71.926(1)$ Å$^3$; Rp = 10.5% for XRD, Rp = 4.6% for ND, final Rp = 9.4%.

**Supplementary Table 2.** Refined structural parameters of chemically delithiated $Li_{0.2}Ni_{0.4}Ru_{0.4}O_2$ based on biphasic $R\bar{3}m$ and $Fm\bar{3}m$ model.

| $Li_{0.2}Ni_{0.4}Ru_{0.4}O_2$: $R\bar{3}m$ | | | | | | |
|---|---|---|---|---|---|---|
| Atom | Wyckoff | x | y | z | Occupancy | Uiso |
| Li(1) | 3 | 0 | 0 | 0.50000 | 0.0000 | 0.01000 |
| Li(2) | 3 | 0 | 0 | 0 | 0.2000 | 0.0249(2) |
| Ru(1) | 3 | 0 | 0 | 0 | 0.4000 | 0.0249(2) |
| Ni(1) | 3 | 0 | 0 | 0 | 0.4000 | 0.0249(2) |
| O(1) | 6 | 0 | 0 | 0.25670(2) | 1.0000 | 0.0054(4) |
| $Li_{0.2}Ni_{0.4}Ru_{0.4}O_2$: $Fm\bar{3}m$ | | | | | | |
| Li(1) | 4 | 0 | 0 | 0 | 0.1000 | 0.02000 |
| Ni(1) | 4 | 0 | 0 | 0 | 0.2000 | 0.02000 |
| Ru(1) | 4 | 0 | 0 | 0 | 0.2000 | 0.02000 |
| O(1) | 4 | 0 | 0 | 0.5000 | 1.0000 | 0.066(2) |

$Li_{0.2}Ni_{0.4}Ru_{0.4}O_2$ lattice parameter: $R\bar{3}m$, 70 mol%, $a = b = 2.90665(9)$ Å, $c = 14.35341(5)$ Å, $\alpha = \beta = 90^o$, $\gamma = 120^o$, $V = 105.020(5)$ Å$^3$; $Fm\bar{3}m$, 30 mol%, $a = b = c = 4.12197(4)$ Å, $\alpha = \beta = \gamma = 90^o$, $V = 70.034(9)$ Å$^3$; Rp = 9.0% for XRD, Rp = 5.0% for ND, final Rp = 8.0%.

**Supplementary Table 3.** Refined structural parameters of $Li_{7/6}Ni_{4/9}Ru_{7/18}O_2$ (LR1) of $Ni^{2+}/Ru^{5+}$ combination based on biphasic $R\bar{3}m$ and $Fm\bar{3}m$ model.

| LR1: $R\bar{3}m$ | | | | | | |
|---|---|---|---|---|---|---|
| Atom | Wyckoff | x | y | z | Occupancy | Uiso |
| Li(1) | 3 | 0 | 0 | 0.5000 | 1.0000 | 0.0342(7) |
| Li(2) | 3 | 0 | 0 | 0.0000 | 0.1700 | 0.0086(4) |
| Ru(1) | 3 | 0 | 0 | 0.0000 | 0.4400 | 0.0086(4) |
| Ni(1) | 3 | 0 | 0 | 0.0000 | 0.3900 | 0.0086(4) |
| O(1) | 6 | 0 | 0 | 0.2523(2) | 1.0000 | 0.0277(8) |
| LR1: $Fm\bar{3}m$ | | | | | | |
| Li(1) | 4 | 0 | 0 | 0.0000 | 0.5850 | 0.00100 |
| Ni(1) | 4 | 0 | 0 | 0.0000 | 0.2200 | 0.00100 |
| Ru(1) | 4 | 0 | 0 | 0.0000 | 0.2950 | 0.00100 |
| O(1) | 4 | 0 | 0 | 0.5000 | 1.0000 | 0.00100 |

$Li_{7/6}Ni_{4/9}Ru_{7/18}O_2$ (LR1) lattice parameter: $R\bar{3}m$, 68.4(3) mol%, $a = b$ = 2.9454(2) Å, $c$ = 14.4281(1) Å, $\alpha = \beta$ = 90º, $\gamma$ = 120º, $V$ = 108.405(2) Å$^3$; $Fm\bar{3}m$, 32.6(3) mol%, $a = b = c$ = 4.16384(4) Å, $\alpha = \beta = \gamma$ = 90º, $V$ = 72.191(2) Å$^3$; Rp = 2.7%.

**Supplementary Table 4.** Refined structural parameters of $Li_{1.2}Ni_{0.4}Ru_{0.4}O_2$ of $Ni^{2+}/Ru^{5+}$ combination based on biphasic $R\bar{3}m$ and $Fm\bar{3}m$ model.

| $Li_{1.2}Ni_{0.4}Ru_{0.4}O_2$: $R\bar{3}m$ | | | | | | |
|---|---|---|---|---|---|---|
| Atom | Wyckoff | x | y | z | Occupancy | Uiso |
| Li(1) | 3 | 0 | 0 | 0.5000 | 1.0000 | 0.0342(6) |
| Li(2) | 3 | 0 | 0 | 0.0000 | 0.2000 | 0.0073(4) |
| Ru(1) | 3 | 0 | 0 | 0.0000 | 0.4000 | 0.0073(4) |
| Ni(1) | 3 | 0 | 0 | 0.0000 | 0.4000 | 0.0073(4) |
| O(1) | 6 | 0 | 0 | 0.2519(1) | 1.0000 | 0.0052(7) |
| $Li_{1.2}Ni_{0.4}Ru_{0.4}O_2$: $Fm\bar{3}m$ | | | | | | |
| Li(1) | 4 | 0 | 0 | 0.0000 | 0.4000 | 0.00100 |
| Ni(1) | 4 | 0 | 0 | 0.0000 | 0.2000 | 0.00100 |
| Ru(1) | 4 | 0 | 0 | 0.0000 | 0.2000 | 0.00100 |
| O(1) | 4 | 0 | 0 | 0.5000 | 1.0000 | 0.00100 |

$Li_{1.2}Ni_{0.4}Ru_{0.4}O_2$ lattice parameter: $R\bar{3}m$, 69.5(4) mol%, $a = b$ = 2.9433(2) Å, $c$ = 14.4204(1) Å, $\alpha = \beta$ = 90º, $\gamma$ = 120º, $V$ = 108.189(2) Å$^3$; $Fm\bar{3}m$, 30.5(4) mol%, $a = b = c$ = 4.1568(1) Å, $\alpha = \beta = \gamma$ = 90º, $V$ = 71.83(6) Å$^3$; Rp = 2.9%.

**Supplementary Table 5.** Refined structural parameters of $Li_{5/4}Ni_{1/3}Ru_{5/12}O_2$ (LR2) of $Ni^{2+}/Ru^{5+}$ combination based on biphasic $R\bar{3}m$ and $Fm\bar{3}m$ model.

| LR2: $R\bar{3}/m$ | | | | | | |
|---|---|---|---|---|---|---|
| Atom | Wyckoff | x | y | z | Occupancy | Uiso |
| Li(1) | 3 | 0 | 0 | 0.5000 | 1.0000 | 0.0342(6) |
| Li(2) | 3 | 0 | 0 | 0.0000 | 0.2500 | 0.0054(2) |
| Ru(1) | 3 | 0 | 0 | 0.0000 | 0.4200 | 0.0054(2) |
| Ni(1) | 3 | 0 | 0 | 0.0000 | 0.3300 | 0.0054(2) |
| O(1) | 6 | 0 | 0 | 0.2548(1) | 1.0000 | 0.00100 |
| LR2: $Fm\bar{3}m$ | | | | | | |
| Li(1) | 4 | 0 | 0 | 0.0000 | 0.6250 | 0.01000 |
| Ni(1) | 4 | 0 | 0 | 0.0000 | 0.1650 | 0.01000 |
| Ru(1) | 4 | 0 | 0 | 0.0000 | 0.2100 | 0.01000 |
| O(1) | 4 | 0 | 0 | 0.5000 | 1.0000 | 0.01000 |

$Li_{5/4}Ni_{1/3}Ru_{5/12}O_2$ (LR2) lattice parameter: $R\bar{3}m$, 75.6(3) mol%, $a = b$ = 2.94330(2) Å, $c$ = 14.4253(1) Å, $\alpha = \beta$ = 90º, $\gamma$ = 120º, $V$ = 108.225(1) Å$^3$; $Fm\bar{3}m$, 24.4(3) mol%, $a = b = c$ = 4.14528(7) Å, $\alpha = \beta = \gamma$ = 90º, $V$ = 71.230(4) Å$^3$; Rp = 2.8%.

**Supplementary Table 6.** Refined structural parameters of $Li_{4/3}Ni_{2/9}Ru_{4/9}O_2$ (LR3) of $Ni^{2+}/Ru^{5+}$ combination based on biphasic $R\bar{3}m$ and $Fm\bar{3}m$ model.

| LR3: $R\bar{3}m$ | | | | | | |
|---|---|---|---|---|---|---|
| Atom | Wyckoff | x | y | z | Occupancy | Uiso |
| Li(1) | 3 | 0 | 0 | 0.5000 | 1.0000 | 0.00100 |
| Li(2) | 3 | 0 | 0 | 0.0000 | 0.3400 | 0.0065(2) |
| Ru(1) | 3 | 0 | 0 | 0.0000 | 0.4400 | 0.0065(2) |
| Ni(1) | 3 | 0 | 0 | 0.0000 | 0.2200 | 0.0065(2) |
| O(1) | 6 | 0 | 0 | 0.2529(1) | 1.0000 | 0.0128(5) |
| LR3: $Fm\bar{3}m$ | | | | | | |
| Li(1) | 4 | 0 | 0 | 0.0000 | 0.6700 | 0.00100 |
| Ni(1) | 4 | 0 | 0 | 0.0000 | 0.1100 | 0.00100 |
| Ru(1) | 4 | 0 | 0 | 0.0000 | 0.2200 | 0.00100 |
| O(1) | 4 | 0 | 0 | 0.5000 | 1.0000 | 0.00100 |

$Li_{4/3}Ni_{2/9}Ru_{4/9}O_2$ (LR3) lattice parameter: $R\bar{3}m$, 79.3(4) mol%, $a = b$ = 2.9407(2) Å, $c$ = 14.4234(9) Å, $\alpha = \beta$ = 90º, $\gamma$ = 120º, $V$ = 108.02(2) Å$^3$; $Fm\bar{3}m$, 20.7(4) mol%, $a = b = c$ = 4.146(2) Å, $\alpha = \beta = \gamma$ = 90º, $V$ = 71.27(1) Å$^3$; Rp = 3.2%.

**Supplementary Table 7. Molar ratio of Li, Ni and Ru obtained by inductively coupled plasma - optical emission spectrometry analysis**. Pristine $Li_{1.2}Ni_{0.4}Ru_{0.4}O_2$ and all designed layered-rocksalt samples ($Li_{7/6}Ni_{4/9}Ru_{7/18}O_2$, $Li_{5/4}Ni_{1/3}Ru_{5/12}O_2$ and $Li_{4/3}Ni_{2/9}Ru_{4/9}O_2$ labeled as LR1, LR2, and LR3, respectively) based on $Ni^{2+}/Ru^{5+}$ combination show ratios close to the designed formula. A gradual change in Ni/Ru ratio is achieved from 1.14 to 0.50 by varying the Li content. For the Rietveld refinement, compositions of $Li_{1.20}Ni_{0.40}Ru_{0.40}O_2$, $Li_{0.50}Ni_{0.40}Ru_{0.40}O_2$, and $Li_{0.20}Ni_{0.40}Ru_{0.40}O_2$ are used for pristine and chemically delithiated samples. The experimental compositions for $Ni^{2+}/Ru^{5+}$ based LR1, LR2, and LR3 samples are used.

| Designed Formula (Sample) | Experimental Molar Ratio |
|---|---|
| $Li_{1.2}Ni_{0.4}Ru_{0.4}O_2$ | Li : Ni : Ru = 1.20(3) : 0.40(3) : 0.40(2) |
| Chemically delithiated #1 | Li : Ni: Ru = 0.50(2) : 0.40(4) : 0.40(3) |
| Chemically delithiated #2 | Li : Ni: Ru = 0.20(5) : 0.40(4) : 0.40(3) |
| $Li_{7/6}Ni_{4/9}Ru_{7/18}O_2$ (LR1) | Li : Ni: Ru = 1.17(3) : 0.44(2) : 0.39(4) |
| $Li_{5/4}Ni_{1/3}Ru_{5/12}O_2$ (LR2) | Li : Ni: Ru = 1.25(4) : 0.33(5) : 0.42(2) |
| $Li_{4/3}Ni_{2/9}Ru_{4/9}O_2$ (LR3) | Li : Ni: Ru = 1.34(3) : 0.22(4) : 0.44(3) |
